# TRPV4 Regulates Breast Cancer Cell Extravasation, Stiffness and Actin Cortex

**DOI:** 10.1038/srep27903

**Published:** 2016-06-13

**Authors:** Wen Hsin Lee, Lee Yee Choong, Naing Naing Mon, SsuYi Lu, Qingsong Lin, Brendan Pang, Benedict Yan, Vedula Sri Ram Krishna, Himanshu Singh, Tuan Zea Tan, Jean Paul Thiery, Chwee Teck Lim, Patrick Boon Ooi Tan, Martin Johansson, Christian Harteneck, Yoon Pin Lim

**Affiliations:** 1 Department of Biochemistry, Yong Loo Lin School of Medicine, National University of Singapore, Singapore; 2 Department of Biological Sciences, Faculty of Science, National University of Singapore, Singapore; 3 Cancer Science Institute of Singapore, Singapore; 4 National University Hospital, Department of Laboratory Medicine, Singapore; 5 Mechanobiology Institute, National University of Singapore, Singapore; 6 Department of Biomedical Engineering, National University of Singapore, Singapore; 7 Duke-NUS Graduate Medical School, Singapore; 8 Respiratorius AB, Lund, Sweden; 9 Department of Pharmacology and Experimental Therapy, Institute of Experimental and Clinical Pharmacology and Toxicology, Eberhard Karls University Hospitals and Clinics, Tübingen, Germany; 10 NUS Graduate School for Integrative Sciences and Engineering, National University of Singapore, Singapore; 11 National University Cancer Institute, National University Health System, Singapore.

## Abstract

Metastasis is a significant health issue. The standard mode of care is combination of chemotherapy and targeted therapeutics but the 5-year survival rate remains low. New/better drug targets that can improve outcomes of patients with metastatic disease are needed. Metastasis is a complex process, with each step conferred by a set of genetic aberrations. Mapping the molecular changes associated with metastasis improves our understanding of the etiology of this disease and contributes to the pipeline of targeted therapeutics. Here, phosphoproteomics of a xenograft-derived *in vitro* model comprising 4 isogenic cell lines with increasing metastatic potential implicated Transient Receptor Potential Vanilloid subtype 4 in breast cancer metastasis. *TRPV4* mRNA levels in breast, gastric and ovarian cancers correlated with poor clinical outcomes, suggesting a wide role of TRPV4 in human epithelial cancers. TRPV4 was shown to be required for breast cancer cell invasion and transendothelial migration but not growth/proliferation. Knockdown of *Trpv4* significantly reduced the number of metastatic nodules in mouse xenografts leaving the size unaffected. Overexpression of TRPV4 promoted breast cancer cell softness, blebbing, and actin reorganization. The findings provide new insights into the role of TRPV4 in cancer extravasation putatively by reducing cell rigidity through controlling the cytoskeleton at the cell cortex.

Breast cancer is the 2^nd^ commonest cancer and the 5^th^ leading cause of cancer-related deaths. Breast cancer kills about 500 000 lives, of which metatastasis is major cause^1^. Adjuvant therapy remains the key pillar in management of metastatic breast cancers (MBCs). There are 4 major subtypes of breast cancer, each distinct in their natural history, molecular portraits, clinical outcomes and responses to treatments^2^,^3^. The normal breast-like and luminal-like subtypes are predominantly ER^+^/PR^+^; HER2 overexpressing breast cancers are predominantly ER^−^/PR^−^ and the basal-like subtype, which is predominantly ER^−^/PR^−^/HER2^−^, also called triple-negative phenotype. Targeted therapies has proven beneficial for treatment of certain breast cancer subtypes. For example, ER^+^/PR^+^ and HER2^+^ tumors account for 75–80% and 15–20% of breast cancer cases and are treated with anti-estrogen and Herceptin therapies, respectively^4^. However, not all patients respond favorably to these targeted therapeutics and subsequent relapse with metastasis is common.

There are much unmet needs for therapeutic intervention at the metastatic stage. Metastasis is a complex process that requires cancer cells to possess multiple traits that endow them with greater motility, plasticity and invasive properties to escape the primary sites as well as to invade and evacuate the blood compartment and lymph vessels in order to colonize distant organs^5^. Each of these steps is believed to involve critical genes. Amongst these are proteins that control the influx/efflux of calcium (e.g. *Orai1* and *STIM1)*^6^,^7^,^8^, epigenetic and transcription factors such as *SATB1*^9^ and *Twist*^10^. Recently, differences in mechanical properties between cancer cells and normal cells were identified by various experimental techniques^11^,^12^,^13^. These studies show that abnormalities in mechanical integrity of cells are associated with cancer pathogenesis and progression. Identification of metastasis-associated genes and further elucidation of the molecular etiology of metastasis are avenues to increase the pipeline of drugs.

The biological models used for metastasis studies thus far do not recapitulate a broad spectrum of the metastatic processes. In contrast, the breast cancer metastasis (BCM) model, which comprises 4 isogenic tumor cell lines 67NR, 168FARN, 4T07 and 4T1, developed by Aslakson *et al*. is increasingly being studied with the advent of high throughput technologies^14^,^15^. It is an attractive model because isogenicity of the model provides a common baseline for comparative studies allowing or enabling the elimination of confounding genetic/epigenetic background variations. The 67NR cancer cell line can form primary local tumors without colonization of distant tissues including blood, lymph nodes and the lungs. Cells of the 168FARN line disseminate from mammary fat pads and can be detected in lymph node but are rarely detectable in other tissues indicating that they can enter the vasculature, whereas extravasation is highly inefficient^14^,^15^. The 4T07 cells are able to spread to the lungs but cannot establish visible metastatic nodules^14^,^15^. Finally, cells of the 4T1 lines are able to complete all steps of metastasis and form visible metastatic nodules in the lungs efficiently^14^,^15^. It is conceivable that mapping the molecular changes of these isogenic cell lines with increasing metastatic potential can lead to the identification of proteins that promote one or more of the key steps in metastasis.

The phosphoproteome is an attractive field for mining cancer discoveries. Phosphoproteomics facilitates the identification of low abundance tyrosine-phosphorylated proteins that is otherwise not possible with conventional expression proteomics. Phosphoproteomics of breast cancer have resulted in the discovery of a novel oncogene^16^,^17^, activating mutations of tyrosine kinase and provided new insights into target-directed therapeutics^18^,^19^. In the present study, we present our data on phosphoproteomic analysis of the BCM model. Transient Receptor Potential Vanilloid subtype 4 (TRPV4), a calcium permeable channel, was found to be strongly correlated with the metastatic status.

TRPV4, a member of the TRP (transient receptor potential) superfamily of cation channels, is the most polymodal TRP channel, being an integrator of both chemical and physical stimuli. Long-standing experimental evidence had shown its physiological relevance to regulation of body osmolarity, mechanosensation, temperature sensing, vascular regulation and, possibly, hearing^20^,^21^. A recent study implicated TRPV4 in tumor angiogenesis, as *Florio Pla* and coworkers reported AA-dependent TRPV4-mediated Ca^2+^ influx selectively drives cell migration via remodeling of the actin cytoskeleton in breast tumor endothelial cells but not in the control cells belonging to human dermal microvascular endothelial cell line^22^. TRPV4 is hence an interesting emerging player in cell migration. Whether TRPV4 contributes to the biology of breast cancer epithelial cells is not known. Herein, the functional roles and mode of action of TRPV4 in migration, invasion and extravasation of breast cancer cells were investigated. Our findings revealed that TRPV4-expressing cancer cells are softer and that TRPV4-conferred cell deformability was associated with actin depolymerization, VASP phosphorylation and inversely correlated with the activation of ERM and Cofilin. Taken together, this study supports a role for TRPV4 in metastasis by regulating cancer cell stiffness and cytoskeleton at the cell cortex.

## Results

### Phosphoproteomics of Breast Cancer Metastasis revealed aberrantly expressed phosphoproteins that are most significantly associated with extravasation

**Figure 1:**
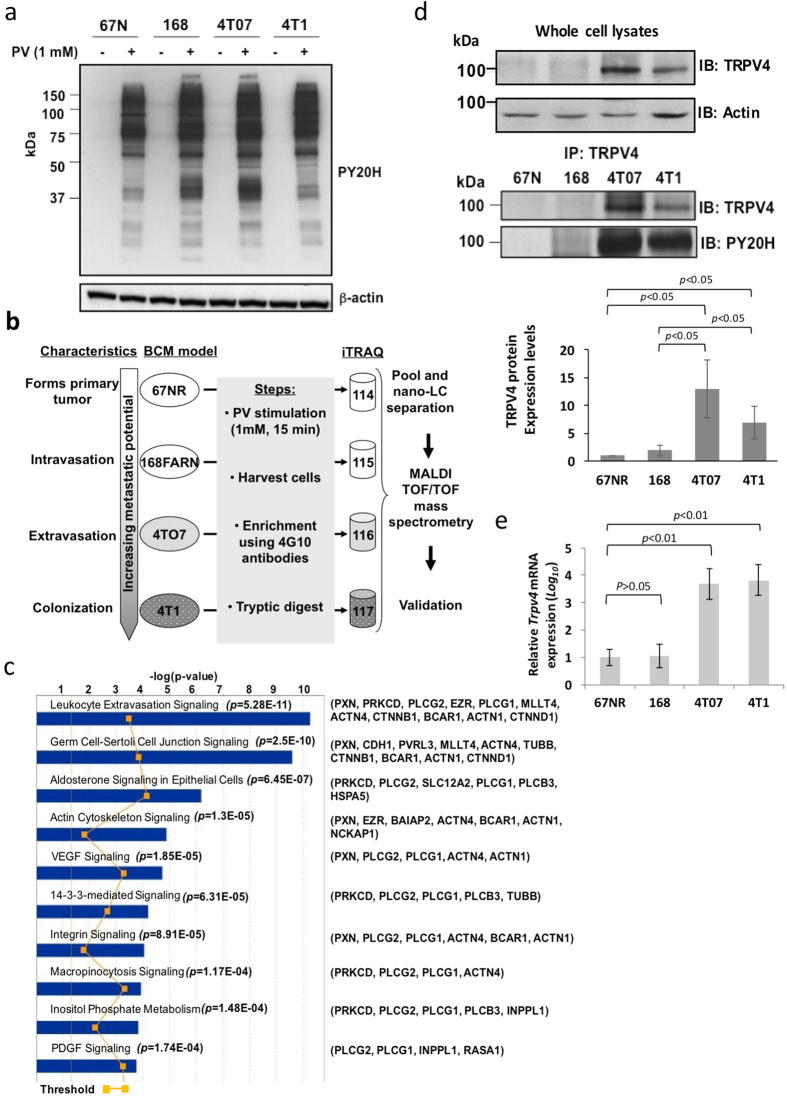
Discovery and validation of TRPV4 as an aberrantly expressed phosphoprotein in metastatic breast cancer cells. **(a)** Pervanadate-induced tyrosine phosphorylation profiles of the cell lines in Breast Cancer Metastasis (BCM) model. **(b)** Schematic diagram showing the workflow of iTRAQ-based experiments to identify phosphotyrosine containing proteins in the Breast Cancer Metastasis (BCM) model. **(c)** Ingenuity Pathway Analysis of the phosphoproteomics gene set. **(d)** Immunoprecipitation shows that TRPV4 is both tyrosine phosphorylated and overexpressed in 4T07 and 4T1 breast cancer cells. Bar chart showing the average values (n = 3, ±s.d.) and *p* values from unpaired Student’s *t*-test are also shown **(e)** Q-PCR showing increased TRPV4 transcript levels across the BCM model. Bar chart was plotted from 3 technical replicates and *p*-values obtained by unpaired Student’s *t*-test.

Since cancer cells accumulate genetic aberrations as disease progresses, we postulate that their phosphoproteome changes as they acquire increasing metastatic potential. Therefore, we examined the phosphotyrosine proteomes of the cell lines across the BCM model following treatment with 1 mM pervanadate for 15 mins which served to enhance the presentation of phosphorylated proteins. Indeed, immunoblotting with anti-phosphotyrosine antibodies (PY20H) revealed that these cell lines possessed distinct patterns (Fig. 1a). Thus, we proceeded to perform phosphoproteomic analysis on the BCM as per the workflow summarized in Fig. 1b. Briefly, tyrosine-phosphorylated proteins were captured using the 4G10 clone of anti-phosphotyrosine antibodies following pervanadate treatment. Captured phosphorylated proteins were then digested using trypsin, the peptides labeled with iTRAQ and subjected to LC-MS/MS. The raw data are provided in Supplementary Table 1 but only proteins whose iTRAQ ratios are statistically significant are listed summarized in Table 1. The iTRAQ ratios reflects the relative amounts of the proteins in 168FARN, 4T07 and 4T01 compared to 67NR. Ingenuity Pathways Analysis (IPA) revealed that the most statistically significant canonical pathway associated with the gene list of 49 proteins was leukocyte extravasation signaling (Fig. 1c), which involves dynamic remodeling of actin, focal and cell adhesion complexes comprising but not limited to the 11 proteins identified in this study. They include paxillin (PXN), β-catenin (CTNNB1), ezrin (EZR), ACTN1 & ACTN4 (F-actin cross-linking proteins). In terms of disease and molecular function, the dataset is most significantly associated with cancer and cell assembly/organization (Supplementary Table 2).

### Up-regulation of TRPV4 protein and mRNA in a murine breast cancer metastasis model

Some proteins in Table 1 are not not known to be associated with metastasis. TRPV4 is particularly interesting, as its expression was barely present in 67NR and 168FARN cells but very high in 4T07 and persisted in 4T1 cells. Supporting data showing the MS^2^ spectra of the 3 TRPV4 unique peptides used for relative quantification by iTRAQ are shown in Supplementary Figure 1. TRPV4 was selected for further investigation since its upregulation correlated with the acquisition of the extravasation trait (possessed only by 4T07 and 4T1). Moreover, while it has been reported to profoundly affect a variety of physiological processes, including nociception, heat sensation and inflammation, TRPV4’s role in cancer is not known.

Immunoprecipitation experiments using anti-TRPV4 antibody and subsequent immunoblot analyses with anti-TRPV4 and anti-phosphotyrosine antibodies validated the differential TRPV4 expression and its tyrosine phosphorylation across the BCM model cell lines (Fig. 1d). The qPCR data also revealed heightened *Trpv4* transcripts in 4T0 and 4T1 compared to 67N breast cancer cells (Fig. 1e). While the increased tyrosine phosphorylation levels of TRPV4 may be due its increased protein expression levels, we cannot rule out that mutation of kinases/phosphatase is not responsible for the increased level of phosphorylated TRPV4.

### *TRPV4* expression correlates with poor outcomes in human epithelial cancers

**Figure 2:**
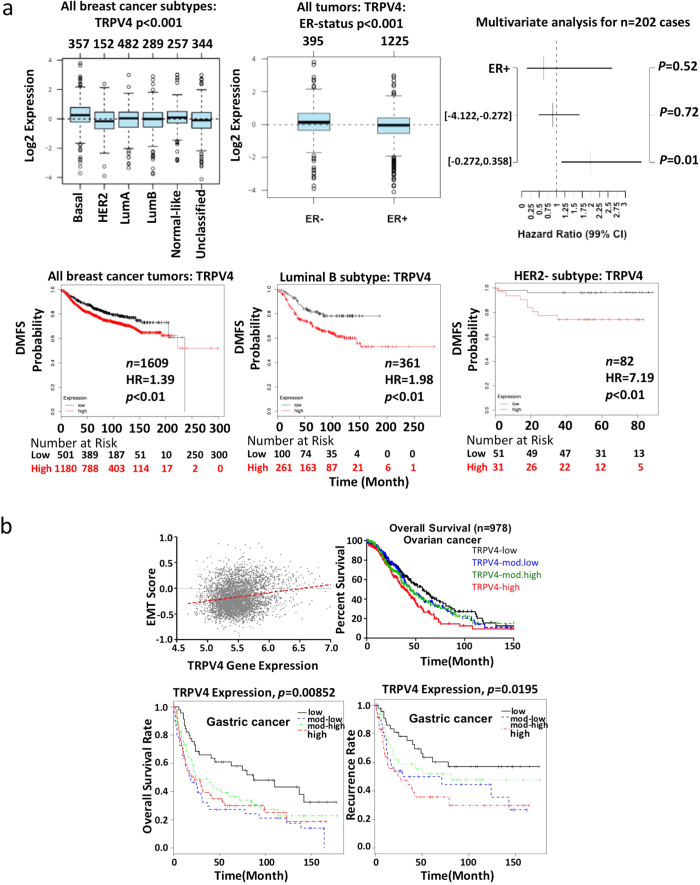
Clinical significance of *TRPV*4 in human epithelial cancers. (**a**) Top: Gene Set Analyses of 1881-sample breast tumors and 51-sample breast cancer cell lines using GOBO. Bottom: Correlation of *TRPV4* expression with DMFS using KMplotter on a total of 361 breast cancers with all breast cancer tumors, luminal B subtype, or HER2 subtype. (**b**) Top left: Correlation of EMT score with *TRPV4* expression in breast tumors. Kaplan Meier Analyses correlating the expression of *TRPV4* with overall and/or disease free survival in ovarian (top right) and gastric tumors (bottom panels).

To examine the significance of TRPV4 to clinical human cancers, we resorted to meta-analysis of public databases through a multifunctional online tool, GOBO (http://co.bmc.lu.se/gobo) that allows different analyses to be performed in an 1881-sample breast tumor data set, and a 51-sample breast cancer cell line set. The datasets are complete with patient information and transcriptomic data generated on Affymetrix U133A microarrays^23^. Using the Gene Set Analysis (GSA)-Tumor application to interrogate the expression of *TRPV4* in 1881 breast tumor samples, *TRPV4* expression was found to be significantly higher in basal subtype compared to the HER2, Lum A and Lum B subtypes of tumors (p < 0.001) (Fig. 2a, top left panel). In addition, *TRPV4* expression was significantly higher in ER-negative compared to ER-positive tumors (p < 0.001) (Fig. 2a, top middle panel). These data supported the notion that *TRPV4* expression is associated with a more aggressive phenotype that correlates with a poorer distant metastasis free survival (DMFS) in a multivariate analyses (Fig. 2a, top right panel). To avoid database-associated biasness, we also performed survival analysis using the Kaplan Meier Plotter (http://kmplot.com/analysis) that base its analysis on gene expression data and survival information from GEO (Affymetrix microarrays only), EGA and TCGA. Samples are split into two groups using a median value across the entire dataset. The results show that *TRPV4* expression correlates significantly with DMFS, n = 1609 with p = 0.0047 and hazard ratio of 1.39. (Fig. 2a, bottom left panel) in the total breast tumors as well as in the patients with lum B (n = 361, HR = 1.98, p < 0.0, bottom middle pane1) and HER-2 negative (n = 82, HR = 7.19, p < 0.01, bottom right panel) subtypes.

In addition, from our DNA microarray studies performed previously on ovarian (Supplementary Materials and Methods) and gastric^24^ cancer, Kaplan-Meier analyses revealed that *TRPV4*-high group correlated with poorer overall and/or disease free survival compared to *TRPV4*-low group (Fig. 2b). Finally, Spearman analysis of Epithelial-Mesenchymal Transition (EMT) score [Supplementary Materials and Methods] and *TRPV4* gene expression revealed that *TRPV4* expression significantly correlates with EMT score (Spearman *Rho* = 0.1534, *p* = 1.4E-22) (Fig. 2b, top left panel).

### TRPV4 is involved in metastatic processes of breast cancer cells *in vitro* and *in vivo*

The above data supported the notion that TRPV4 plays a role in cancer progression. TRPV4 contributes to the regulation of normal epithelial cell permeability by modulating tight junction proteins^25^. Furthermore, the intracellular localization of TRPV4 shifted from intracellular compartments to the plasma membrane during epithelial-mesenchymal transition (EMT). This may have implications in cancer cell metastasis^26^. Hence, we proceeded to perform wound healing, chemotaxis, matrigel invasion and transendothelial migration assays to obtain direct evidence for the role of TRPV4 in metastasis.

**Figure 3:**
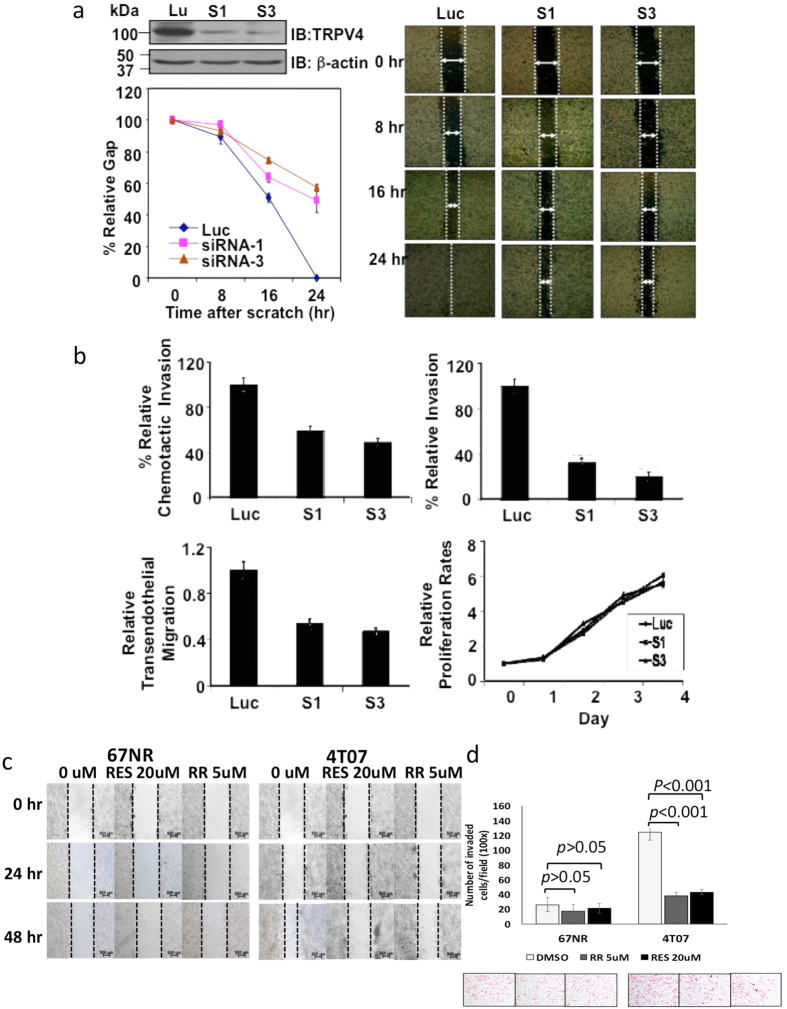
*In vitro* cell-based assays following *Trpv4* knock down. (**a)** Top left panel shows effective silencing of TRPV4 expression. Bottom left and right panels show that *Trpv4* siRNA inhibited the migration of 4T07 cells. **(b)** *Trpv4* siRNAs inhibited the chemotaxis, invasion, transendothelial migration but not proliferation of 4T07 cells. For all, except proliferation, knock down of *Trpv4* produced statistically significant effect on the cellular processes studied, *p* < 0.05. **(c)** Shows the inhibitory effects of RR and RES on cell migration and **(d)** invasion of 4T07 cells but not 67NR cells. Average values (n = 3, ± s.d.) from three biological repeats and *p* values from unpaired Student’s *t*-test are shown.

4T07 was the cell line of choice in the BCM model to study migration, invasion and extravasation following RNAi-based loss of function studies since 4T07 cells possess various traits of metastasis including the extravasation step^14^,^15^. Besides, TRPV4 is highly expressed in this cell line. Three *Trpv4*-specific siRNAs were screened and two of the siRNAs, S1 and S3 that most effectively knocked down TRPV4 expression were used for further studies (Fig. 3a, top left panel). Subsequent *in vitro* cell-based assays showed that TRPV4 knockdown consistently diminished wound-healing (by 50–60%), chemotaxis (by 40–50%), matrigel invasion (by 70–80%) and transendothelial migration (by 50%) (Fig. 3a,b). In contrast, such an inhibitory effect on cell migration were not seen when TRPV4 was siRNA-silenced in the 67NR cells which has low levels of TRPV4 expression (Supplementary Figure 2). The effects of *Trpv4* silencing on these metastatic processes were not due to a decrease in cell number/viability since silencing *Trpv4* expression had no significant effect on cell proliferation compared to control cells (Fig. 3b, bottom right panel). To further elucidate the ion channel function of TRPV4 in cancer metastasis, we employed Ruthenium Red (RR) and RES019–29 (RES) compound to inhibit the functions of TRPV4. RR has been commonly used as a TRPV4 inhibitor to block its ion channel function and for over the decades. Besides RR, RES019–29 (RES) compound was found to have selectivity for TRPV4 (Supplementary Figure 3). Both inhibitors were found to effectively suppress the migration (Fig. 3c) and invasion (Fig. 3d) of the 4T07 cells. In contrast, the effects were barely seen in the 67NR cells which has undetectable levels of TRPV4.

**Figure 4:**
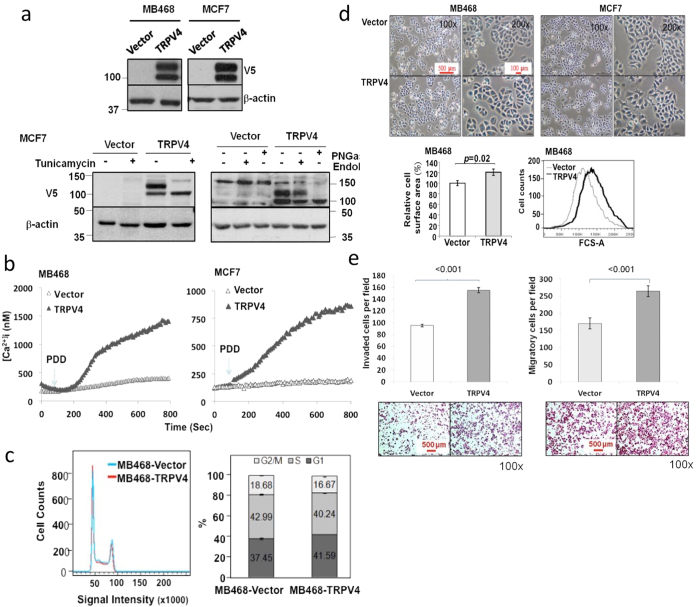
Effects of exogenous TRPV4 on human breast cancer cellular processes. (**a)** Expression of wild-type human TRPV4 in stably retroviral-transduced breast cancer cell lines. Upper panel: Immunoblotting of exogenous TRPV4 and endogenous Actin (loading control) in whole-cell lysates. Lower panel: Immunoblotting of TRPV4 following Tunicamycin treatment (5 ug/mL), PNGaseF and Endo-H digestion (500 U/reaction). (**b)** Calcium imaging of TRPV4 stable transductants following treatment with 10 μM of 4α-PDD. (**c**) FACS analysis of MB468-TRPV4 stable transductants. Left panel: after gating on the live cells, single cells were gated using width and area parameters. Right panel: Histogram showing the percentages of cells in G1, S, and G2M phases. Average values (n = 3, ±s.d.) from two biological repeats and *P* values from unpaired Student’s *t*-test are shown. (**d)** Physical examination of TRPV4 stable transductants. Upper panel: Phase-contrast micrographs of the cell morphology of MB468 and MCF7 transduced with pBABE or pBABE-*TRPV4* were obtained at sub-confluent density grown in standard medium containing 10% serum. Bottom left panel: relative cell surface area of control and TRPV4 stable transductants; bottom right panel: size distribution of control and TRVP4 stable transductants based on FSC-A data from FACS analysis and *P* values from unpaired Student’s *t*-test are shown (**e)** Effects of TRPV4 on the matrigel invasion and chemotaxis of MB468 cells transduced with pBABE or pBABE-*TRPV4* retroviral particles using the Boyden chamber assays. Cells that migrated or invaded through the barrier were stained with hematoxylin/eosin and counted (for invasion assay) or subject to colormetric measurements (chemotaxis assay) in three independent experiments. Data are expressed as migrated/invaded cells per field (n = 3, means ± s.d.), *P* values from unpaired Student’s *t*-test are shown.

The above RNAi-based loss of function studies in mouse cancer cells support the idea that TRPV4 plays a role in metastasis. To determine whether this is true in human breast cancer cells, we performed gain-of-function overexpression studies. (MB468) and MCF7 breast cancer cell lines were used since they display low migratory ability *in vitro*. Exogenous V5-tagged human TRPV4 presented itself as 2 species, with the higher MW form being glycosylated since this species is sensitive to glycosylation inhibitors PNGaseF and EndoH (Fig. 4a). Such dual species of of TRPV4 observed during exogenous expression in human BC cells were not apparent in mouse BC cells. The reason for this observation is uncertain. Exogenous TRPV4 was functional with respect to the ability to mediate calcium flux (Fig. 4b).

First, we confirmed if TRPV4 overexpression has a role in human cancer cell proliferation by performing FACS analysis. Overexpression of TRPV4 did not apparently affect cell cycle of MB468 (Fig. 4c) and MCF7 (Supplementary Figure 4) stable transductants. This is consistent with the previous observation that knock-down of TRPV4 did not affect proliferation of mouse 4T07 breast cancer cells. However, MB468 but not MCF7 TRPV4 transductants cells were observed to have a larger size microscopically compared to control cells (Fig. 4d, upper panels). Analysis of the cell surface area using ImageJ software further supports the observation that TRPV4-overexpressing MB468 cells were larger than the control cells by 20% (Fig. 4d, bottom left). This is supported by a shift in the forward scatter (FSC) fluorescence intensity of the MB468-TRPV4 cells compared to control cells during FACS analysis (Fig. 4d, bottom right). It is unclear what bearing this has but this might reflect the role of TRPV4 in regulating cellular osmotic pressure and swelling.

**Figure 5:**
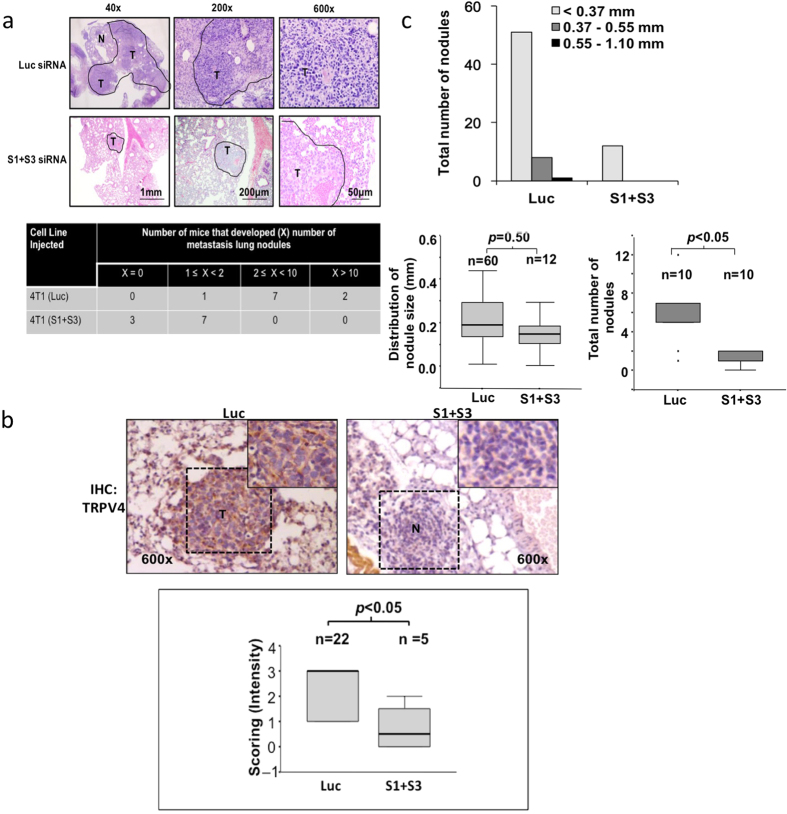
(a) Effect of *Trpv4* knock down on metastasis of 4T1 cells. Top panel: H&E staining of lung tissue sections from mice injected with 4T1 cells transfected with control or *Trpv4*-specific siRNA. ‘T’ indicates tumor nodules and ‘N’ indicates normal lung tissues. Three magnifications (40x, 200x and 600x) of the representative H&E images (n = 10 for each groups). Bottom panel: Table showing the number of mice in the control or *Trpv4*knocked-down condition that possessed the categorical numbers of nodules. **(b)** Upper panel: Representative IHC images showing the expression of TRPV4 in the nodules of lungs of mice from control and *Trpv4*-knocked down conditions. Lower Panel: Box plots showing the expression of TRPV4 in the metastatic nodules in the lungs from mice in the control and *Trpv4*-knocked down conditions. **(c)** Top panel: The size of all the nodules in lungs of every mouse in control and *Trpv4*-knocked down conditions were measured and the numbers of nodules within 3 size groups were plotted. Bottom left panel: Box plots showing the distribution of nodules size in the mice within the control and *Trpv4*-silenced conditions. ‘n’ refers to the sample size of the nodules in each condition. Bottom right panel: Box plots of the total number of nodules in each mouse within the control and *Trpv4*-silenced condition. ‘n’ refers to sample size of mice.

Next, we investigated the effect of exogenous expression of TRPV4 on human breast cancer metastasis. While TRPV4 overexpression did not alter the invasive and chemotactic ability of the MCF7 (data not shown), invasiveness and chemotaxis of TRPV4-overexpressing MB468 cells were increased by about 50–60% compared to the vector control (Fig. 4e). TRPV4 overexpression did not promote the transendothelial migration ability in either MCF7 or MB468 cells (data not shown). To obtain a more physiological and an overall insight into the impact of TRPV4 in extravasation, metastasis and formation of visible metastatic nodules in the lungs, we performed tail vein injection of control and *Trpv4* knocked-down 4T1 cells into SCID mice. 4T1 cell line is suitable for this purpose as it expresses high level of TRPV4, is originally reported to be capable of the entire spectrum of metastatic process from invasion to colonization of distant sites and is commonly used for *in vivo* metastasis studies^6^,^10^,^14^,^27^,^28^,^29^. Cells were introduced via tail vein into the mice following TRPV4 knock down. After 7 days of injection, mice from each group were sacrificed and lungs harvested to measure the colonization of metastatic cells by staining the tissues with hematoxylin and eosin (H&E) (Fig. 5a, upper panel). The number of mice that showed the various categorical numbers of nodules in each condition is shown in Fig. 5a, bottom panel. It is apparent that less mice developed nodules but when they did, had lower number of nodules in the lungs when TRPV4 was silenced compared to control. As illustrated in Fig. 5b, immunohistochemistry of the lung sections revealed that the expression of TRPV4 in the nodules formed in the lungs of mice injected with *Trpv4* knocked-down 4T1 cells was significantly lower compared to those from control mice (p < 0.05). Fig. 5c top panel shows the number of size-classified metastatic nodules that formed in mice injected with *Luc* control or *Trpv4* knocked-down 4T1 cells. The results showed that silencing of *Trpv4* expression reduced the number of lung metastasis by approximately 80% (60 versus 12 nodules). There was no statistically significant difference in the size of the nodules formed (Fig. 5c, bottom panels). This is consistent with the earlier observation that TRPV4 does not affect cancer growth/proliferation. A complete list of nodules counting and IHC results are provided in Supplementary Table 3. In summary, our data show that TRPV4 contributes to breast cancer metastasis.

### TRPV4 regulates cell stiffness

**Figure 6:**
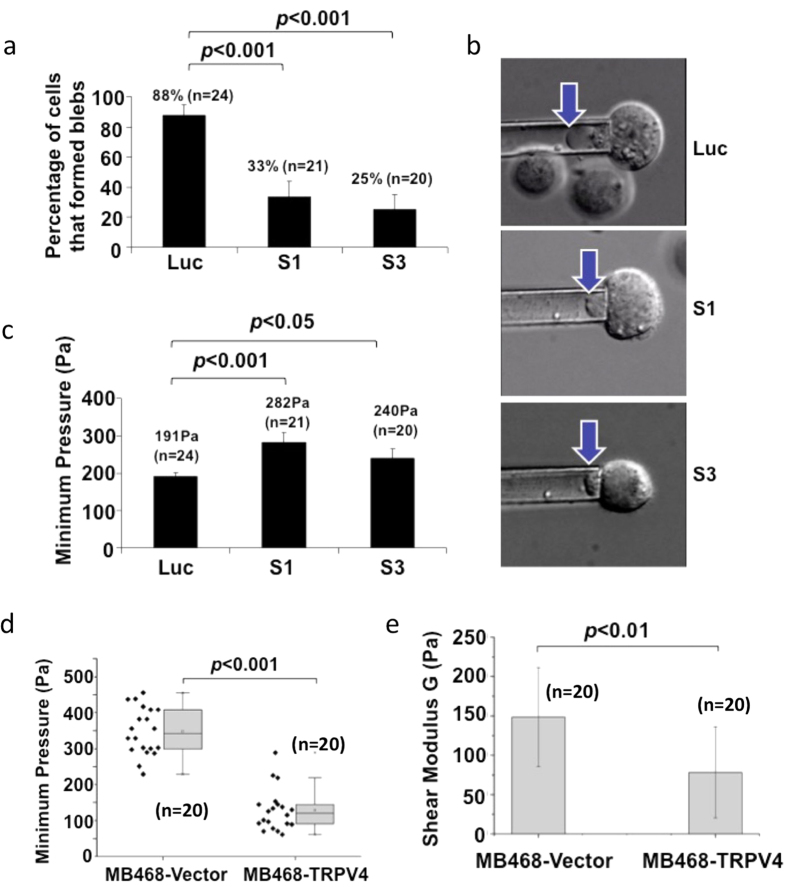
Micropipette aspiration experiment to measure cancer cell rigidity following *Trpv4* knock down or overexpression in 4T07 and MB468 cells, respectively. **(a)** The percentage of 4T07 cells that formed blebs before and after *Trpv4* silencing. **(b)** Arrows show that bleb formed in control cells but not cells transfected with S1- and S3- *Trpv4*-specific siRNAs. The minimum pressure at which bleb developed in **(c)** 4T07 cells and **(d)** MB468 cells were plotted. **(e)** The shear modulus (*Pa*) in the MB468 cells was determined from using the linear elastic solid model. *P* values from unpaired Student’s *t*-test are shown.

To facilitate extravasation, metastatic cells would acquire several traits including but not limited to the ability of the cells to adhere/dock to molecules on the surface of endothelial cells and to traverse endothelial cell lining without causing damage to the cancer cells. The latter invariably involves actin cytoskeleton remodeling, production of blebs or lamellipodia. As TRPV4 is able to mediate Ca^2+^ fluxes, which are well known to play role in these processes^8^, and to understand the cellular mechanism of TRPV4’s function, we hypothesized that transendothelial migration and metastasis were promoted via TRPV4-conferred cellular “softness”. Therefore, we performed micropipette aspiration experiments and monitored the formation of membrane bleb in *Trpv4* knocked-down 4T07 cells versus control cells. Cells were suspended in growth media and subjected to negative pressure, allowing individual cells to be aspirated into the opening of the micropipette by the suction forces. Blebs are formed when a portion of the cell membrane detached from the under-lying cortex under the influence of a cytosolic pressure^30^. It was observed that the number of cells that formed blebs at the end of the assay was significantly lower in *Trpv4* knocked-down cells compared with control cells (Fig. 6a). Representative images showing the formation of a bleb in control cells but not in *Trpv4* knocked-down cells are shown in Fig. 6b. Moreover, the minimum pressure required for the formation of blebs in control cells was also significantly lower compared to that required for *Trpv4* knockdown cells (Fig. 6c). Raw data are provided in Supplementary Table 4. Representative videos of control, S1- and S3- *Trpv4* knocked-down cells subject to micropipette aspiration assays are provided in this website (http://www.med.nus.edu.sg/bch/pi/lyp/video. php, password: video).

In accord with the loss of function studies in 4T07 murine breast cancer cells, gain-of-function studies by overexpression of TRPV4 in MB468 showed enhanced cell blebability. The pressure at which bleb initiation occurred in MB468-TRPV4 cells was significantly lower than that in the control group (Fig. 6d). Furthermore, the effect of TRPV4 on cell stiffness was estimated by measuring the shear modulus (G) of the cell by micropipette aspiration technique (details mentioned in materials & methods section). As shown in (Fig. 6e), TRPV4 overexpression reduced the shear modulus (Pa) by > 2 times as compared to the control, suggesting lower force was required to deform the cells. Collectively, the data support the notion that physical “elasticity” is a potential mechanism through which TRPV4 confers metastatic potential to cancer cells – a process that may involve serverence of the plasma membrane with the cytoskeletal proteins in the cell cortex region.

### TRPV4 accelerates actin dynamics and downregulates cytoskeleton-associated proteins in the cell cortex

**Figure 7:**
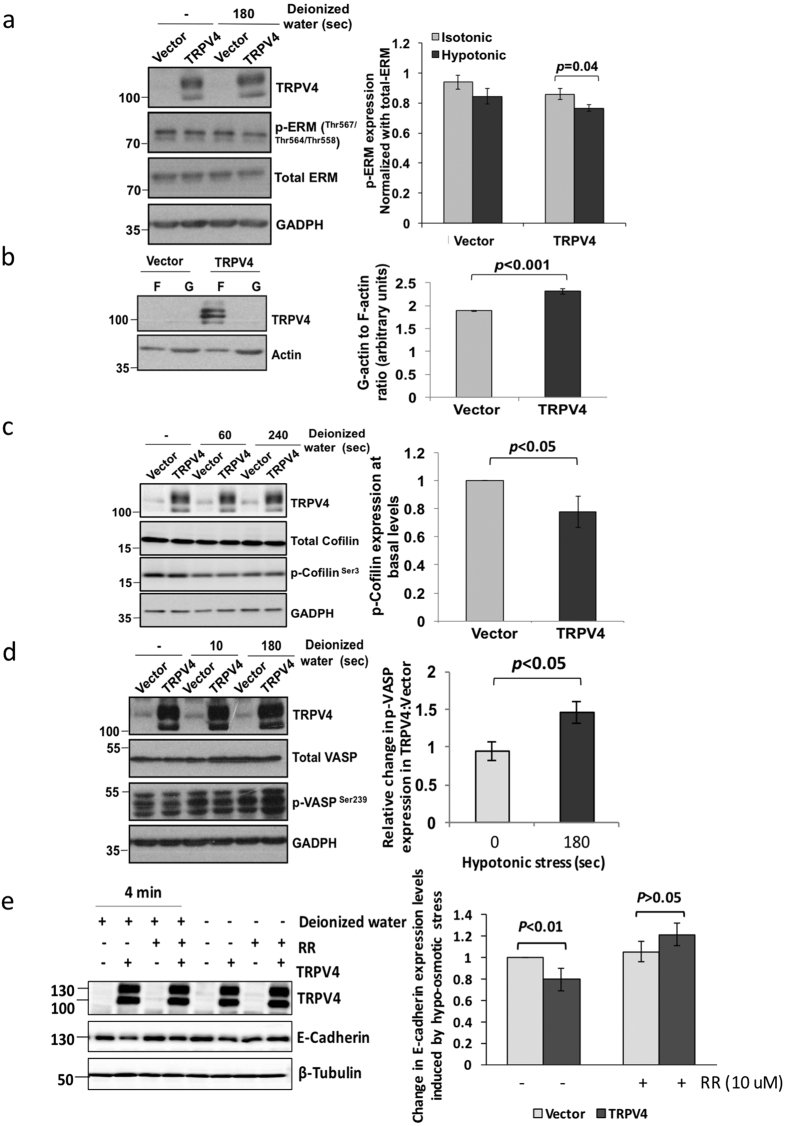
TRPV4 overexpression affected the cytoskeleton network compared to the control. (**a**) Lysates from control or stable TRPV4 MB468 transductants untreated or treated with deionized water for 3 min were immunoblotted with the indicated antibodies. Data shown are mean ± s.d. (*n* = 3) (**b**) Insoluble (contains F-actin) and soluble fractions (contains G-actin) isolated from the total lysates were probed for Actin. Data shown are mean ± s.d. (*n* = 4). (**c**) Lysates from control or stable TRPV4 MB468 transductants were immunoblotted with the phospho-Cofilin or total Cofilin antibodies. (**d**) Lysates from MB468-Vector and MB468-TRPV4 cells not treated or treated with deionized water were probed for total VASP and phospho-VASP antibodies. Protein expression levels were normalized and quantified as shown by the bar chart. GAPDH were used as a loading control. Average values from three biological repeats (n = 3, ±s.d.) and *p* values from unpaired Student’s *t*-test are shown. (**e**) MB468-Vector and MB468-TRPV4 cells were not pre-treated or treated with RR for an hour before the replacement of the media with deionized water or fresh growth medium. Densitometry was performed. Protein expression levels were quantified and presented as relative change in E-cadherin expression levels in the various conditions compared to their respective untreated samples. *β*-Tubulin were used as a loading control. Average values from three biological repeats (n = 5, ±s.d.) and *p* values from unpaired Student’s *t*-test are shown.

The plasma membrane-cytoskeleton interface is occupied by linker proteins that physically couple the membrane to the cytoskeleton. For instance, the ERM proteins (ezrin, radixin and moesin) are known to be involved in cell mechanics and plasma membrane-actin cortex interaction^31^,^32^. Upon phosphorylation and activation, these proteins can bind both to polymerized actin and integral membrane proteins, leading to reduced blebbing^33^. Thus, the rigidity of the cell is likely to be dependent on the density and strength of the juxtamembrane scaffolds formed by the ERM linker proteins. To investigate the molecular mode of action of TRPV4 on the regulation of cell cortex proteins during TRPV4-conferred cellular “softness”, we examined the ERM proteins in control and TRPV4-expressing cells. In isotonic condition, the level of phosphorylated ERM, shown as a doublet band, was 5–10% lower in in MB468-TRPV4 compared to control cells (Fig. 7a). However, when control and MB468-TRPV4 cells were subjected to stimulus in the form of hypo-osmotic stress, the decrease in the phospho-ERM levels between the MB468-TRPV4 and control cells exacerbated to 10–20%. Decrease in ERM phosphorylation has been linked to blebability of the cells and reduced actin cortex strength, which supports our observation^34^. Therefore, our observations indicate a weaker link between F-actin and trans-membrane proteins (Fig. 7a), hence weaker bond strength of the cell membrane-cortex in TRVP4-expressing cells compared to control cells.

As TRPV4 has been shown to bind directly to cytoskeletal proteins, including actin, tubulin and neurofilament^35^, we hypothesized that TRPV4 could alter the biomechanical properties of the cells via regulating the actin network. Hence, we investigated whether TRPV4-induced softness was associated with actin depolymerization. High-speed centrifugation was used to separate the G- and F-actin pools from cell lysates, followed by quantitative Western blot analysis to assess the ratio of G- to F-actin. There was a statistically significant 22% increase in the average G to F actin ratio in TRPV4-transfected cells compared to control cells (2.31 versus 1.89, n = 4) (Fig. 7b). Cofilin is one of the actin-depolymerizing factors that directly regulate actin dynamics^36^,^37^. Phosphorylation of cofilin suppresses its actin-depolymerizing functions^38^,^39^. When the phosphorylation status of cofilin was examined, phospho-Cofilin expression level in MB468-TRPV4 was 1.45 fold less ± 0.04 (n = 3) compared to vector transfected cells (Fig. 7c). VASP promotes actin polymerization by restricting actin filament capping while VASP phosphorylation at S239 impaired actin filament formation^40^. No difference in VASP phosphorylation was observed between vector control and TRPV4-trasnfected cells in the absence of stimulus. When cells were subject to osmotic stress, VASP phosphorylation progressively increased moderately in vector control cells but drastically in TRPV4-transfected cells (Fig. 7d). This may explain the earlier observation that TRPV4-transfected cells had less F actin compared to vector control cells. Lastly, we examined the stability of E-cadherin in association with TRPV4 under the hypo-osmotic conditions since E-cadherin is an adherens junction protein plays an important role in cytoskeletal organization and adhesion. Figure 7e shows that at the basal level, reduction in E-cadherin expression was detected in MB468-TRPV4 as compared to the vector control. And a further decrease was seen under the hypo-osmotic conditions as deionized water exacerbated the instability of E-cadherin proteins when TRPV4 was being overexpressed. Lastly, we asked whether functionality of TRPV4 ion channel may have contributed to the changes observed in apical protein expression levels by using RR. The data shows that TRPV4 inhibitor could prevent the loss of apical proteins at the basal levels (Fig. 7e). In the hypo-osmotic condition, a partial restoration of E-cadherin expression by RR treatment was observed (Fig. 7e), suggesting that the hypo-osmotic effects triggered by deionized water was partially mediated via TRPV4 ion channel. Collectively, the data suggest that TRPV4 promotes cancer cell extravasation/transendothelial migration by reducing cell stiffness through regulation of the cell cortex/cytoskeleton proteins.

## Discussion

TRPV4 is a member of the TRP (transient receptor potential) superfamily of cation channels, which have been implicated in human diseases and cancer. Dysregulation of the expression of TRPM1, TRPM8, TRPC6, TRPV1, TRPV2 & TRPV6 have been observed in prostate, ovarian, breast, liver and bladder cancers^41^,^42^,^43^. The role of TRPV4 in epithelial cancer cells has not been reported. Here, we show that TRPV4 protein and transcript is overexpressed in an experimental model of breast cancer metastasis and its overexpression is associated with the acquisition of extravasation traits by breast cancer cells. Clinically, higher *TRPV4* transcript expression is associated with the more aggressive breast cancer subtypes (e.g. ER- and basal) and correlates with a poorer distant metastasis free survival of patients with breast, ovarian and gastric cancer – implying a wide role of TRPV4 in solid epithelial cancers. Gain and loss of function studies in murine and human breast cancer cells supported a role of TRPV4 in metastasis.

In addition to formation of lamellipodia or filopodia, cell migration and invasion can also be driven by a very different mechanical process - blebbing, which is known to be associated with amoeboid movement in invasive cancer cells. Blebs are specialized rounded membrane protrusions which were initially identified as morphological feature of apoptosis^44^,^45^. Only until recent decades, a collection of data demonstrates bleb formation as a special type of cell motility in the cells due to increase in intracellular pressure and these cells showed no signs of apoptosis^46^. Plasma membrane blebbing is now believed to be associated with EMT^47^ and can be induced by oncogene such as *c-MET* leading to amoeboid cell motility and invasion^48^. Another study shows that breast cancer cells, invading through 3D collagen under conditions in which matrix degradation is blocked, used an amoeboid mode of invasion with the formation of bleb-like constriction rings^49^. Interestingly, both the expression and activation of TRPV4 has been shown to induce striking morphological changes affecting lamellipodial and filopodial structures^35^.

Amoeboid movement via bleb formation is known to be dependent on cellular deformability (degree of stiffness), a parameter we examined by micropipette aspiration. We concede that the blebs induced in this study were produced by an external physical force, we nevertheless translated these observations into biomechanical measurements through the use of a linear elastic solid model. The model was based on a uni-directional approximation of the blebbing dynamics, to get insights into in the rigidity of the cells. In both murine and human models, TRPV4 expression was found to correlate with decreased shear modulus and increased deformability. Reduced association between the cortex and the membrane as indicated by less phospho-ERM could have promoted blebbing^50^. More recently, Thodeti *et al*.^51^ demonstrated that mechanical forces that activate TRPV4 and physically deform ECM can guide capillary cell reorientation. The authors showed that cyclically stretching capillary endothelial cells adherent to flexible ECM substrates activate TRPV4, that in turn, stimulate PI3K-dependent activation and binding of additional beta-1 integrins, which promotes cytoskeletal remodeling and cell reorientation during angiogenesis. Another study by Alessandri-Haber N *et al*.^52^ showed that alpha2beta1 integrin and Src tyrosine kinase, which have been implicated in mechanical transduction, are important for the development of mechanical hyperalgesia, and that their contribution requires TRPV4. It is possible that TRPV4 physically and functionally collaborate with integrins in cancer cells and capable of triggering the signal transduction cascades that leads to the cell mobility. Other mechanisms that we have not investigated include myosin II-induced actomyosin contraction that could drive the formation of blebs by increasing hydrostatic pressure, leading to focal rupture of the actin cortex^50^. It would be interesting to further investigate how mechanosensitive TRPV4 modulates cytoskeletal remodeling by using the immunofluorescence tool to study TRPV4-induced actin organization, and localization of cortical proteins (e.g. ERM and myosin) during bleb expansion and retraction.

In addition to ERM cell cortex proteins, actin polymerization is another factor that influence mechanical cell stiffness. TRPV4 overexpression in MB468 increased the ratio of soluble G-actin over filamentous F-actin, reiterating the role of TRPV4 in reducing cell stiffness. This was concomitant with increased level of phosphorylated VASP, presumably resulting in less restriction of actin filament capping, thereby resulting in less actin polymerization. Moreover, a decrease in cofilin phosphorylation, which inhibits its actin depolymerization function, in the MB468-TRPV4 cells could have “softened” the cells by weakening the actin cortex. Overall, overexpression of TRPV4 in MB468 cells conferred physical softness to breast cancer cells via decreasing scaffolding by cell cortex proteins and increasing G to F actin ratio. The findings provide, for the first time, new insights into the molecular and cellular basis of TRPV4 function in cancer metastasis. These observations are consistent with the reported roles of TRPV4 in regulating normal epithelial cell permeability and volume through its action on actin microfilament dynamics^25^,^53^.

It is conceivable that as cancer cells get progressively more invasive, they display softer mechanical characteristics that facilitate cell deformation and shape changes suitable for metastatic population. This model is supported by our findings as well as that of others which showed that highly metastatic epithelial cells (e.g. ovarian, lung and breast duct) had reduced stiffness that is associated with actin cytoskeleton remodeling as compared to less invasive cells^12^,^13^,^54^,^55^,^56^. However, increased stiffness and decreased deformability of cancer cells in the prostate malignancies have also been reported^57^,^58^,^59^. Such apparent controversy could be a result of several factors including the cancer cell types studied, oncogenes involved, the conditions in which the assays were performed in and the techniques used. Changes in stiffness or elasticity of pro-metastatic cancer cells had been predominately studied in isolated cells or *in vitro* cell culture systems. Recently, *Fenner et al*. directly link relative stiffness of tumors to cancer progression by using a mouse model of metastatic breast cancer together with *ex vivo* measurements of bulk moduli of freshly excised, intact tumors^60^. This approach reproduces surgical resection of a mammary tumor with subsequent local recurrence and metastasis. In support of our findings, this paper showed that more compliant tumors were associated with more frequent, larger local recurrences and more extensive metastaese than mice with relatively stiff tumors.

Could the understanding of the mode of action of TRPV4 shed light on its effect on the metastatic processes examined? TRPV4 overexpression promoted invasion and migration of MB468 but not MCF7 cells. Furthermore, contrary to the loss-of-function studies in 4T07, TRPV4 overexpression did not increase the transendothelial migration in MB468. 4T07 are fibroblastic and highly invasive whereas MB468 cells are epithelial in nature. It is conceivable that while TRPV4 is required, it may not be sufficient to drive metastatic processes in some cells. This is plausible since the key mode of action of TRPV4 identified in this study is in regulating cell actin cortex and stiffness. Nevertheless, this does not rule TRPV4 out as a potential drug target. It may also be interesting to study the role of TRPV4 phosphorylation in actin cortex control and cell softness.

TRPV4 is overexpressed in the basal subtype of breast cancer, most of which are aggressive and triple-negative in nature. TRPV4 is viable drug target since it is a cell surface cation channel that can be inhibited by small molecules such as Ruthenium Red^25^. TRPV4 may be exploited as a drug target for management of triple negative, metastatic breast cancers. However, TRPV4 does not seem to be required for cancer cell growth/proliferation. A rational cancer therapy strategy would require combining anti-TPRV4 inhibitors (to potentially block metastasis) with cytotoxic drugs (to kill cancer cells). Anti-TRPV4 antibody-drug conjugate may also be designed to target metastatic cancer cells with improved specificity and efficacy.

In conclusion, a novel role for TRPV4 in metastasis has been established. Regulation of cell stiffness and cell cortex dynamics are putative modes of actions through which TRPV4 promotes cancer cell extravasation. TRPV4 may be a viable drug target for management of metastatic breast cancers.

## Materials and Methods

### Reagents

TRPV4 rabbit polyclonal antibodies were generated in Dr Christian Harteneck’s laboratory using a TRPV4-derived peptide sequence (N terminus-CENPHKKADMRRQDS-C-terminus). 4α-alpha-phorbol 12,13-didecanoate and Ruthenium Red were from Merck KGaA (Darmstadt, Germany). RES019-29 was provided by Martin Johansson (Respiratorius AB, Sweden). The rest of the reagents are detailed in Supplementary Materials & Methods.

### Cell lines and lysis

The breast cancer metastasis model cell lines (67NR, 168FARN, 4TO7 and 4T1) were obtained from Dr. Fred Miller at the Barbara Ann Karmanos Cancer Institute (Detroit, MI). The culture conditions were as previously described^14^. Culture conditions for other commercially available cell lines and the lysis method are provided in Supplementary Materials & Methods.

### LC-MS/MS of 4G10-purified phosphoproteins – detection and relative quantification

Serum-starved cells were treated with 1 mM pervanadate (PV) for 15 min to increase the representation of tyrosine phosphorylated proteins and lysed. Preparation of 1 mM PV was as previously described^61^. From big scale preparations of cell lysates, one portion was used for phosphoproteomics analysis and a smaller portion retained for subsequent validation. For the former purpose, sixty milligrams of total protein from each cell lines were used for purification via 4G10 anti-phosphotyrosine antibodies-based immunoaffinity purification. This was performed and processed for iTRAQ labeling as previously reported^16^. The samples were then pooled and cleaned-up by the cation exchange cartridge provided in the kit. Two biological replicates were prepared and the samples desalted, lyophilized and analyzed using MALDI-TOF-TOF (see Supplementary Materials & Methods for details).

### TRPV4 knock down and overexpression

Mouse *Trpv4*-specific siRNA oligonucleotides were purchased from Invitrogen (Carlsbad, CA) and the siRNA sequences are as following: *Luciferase* GL2: 5′-CGUACG CGGAAUACUUCGA-3′; *Trpv4* siRNA1: 5′-AGAAGCAGCAGGUCGUACAUCUUGG-3′; *Trpv4* siRNA3: 5′-AAACUUGGUGUUCUCUCGGGUGUUG-3′. Transduction protocols for *TRPV4* overexpression in human breast cancer cells are provided in Supplementary Materials & Methods.

### *In vivo* model

The protocol for the xenograft study was reviewed and approved by the Institutional Animal Care and Use Committee (IACUC) of the National University of Singapore in compliance with international guidelines on the care and use of animals for scientific purpose. All methods were carried out in accordance with the relevant guidelines. 4T1 (1 × 10^6^) cells in 150 μL PBS were injected into the tail vein of eight-weeks-old female severe combined immunodeficiency mice. After 5, 7, 10 and 14 days of injection, mice were euthanized and examined for the metastasis of the lungs. The optimal duration of the assay was determined to be 7 days. To count the number of metastatic nodules, mouse lungs were collected and fixed with 10% neutral buffered formalin (Sigma) for 16 hr at 4 °C, processed by Thermo Shandon tissue processor and embedded in paraffin. Sections were warmed in a 60 °C oven, dewaxed in three changes of histoclear and passaged through graded ethanol (100%, 95%, and 70%) before a final wash in double distilled H_2_O. The nodules size was recorded for each Hematoxylin and Eosin (H&E)-stained section using the Olympus BX-41 light microscope (Center Valley, PA) at high-power field (HPF; x400). The maximum diameter of viable nodule was calculated by summing the largest uni-dimensional diameter of each fragment of nodule using the Olympus BX-41 microscope and the micrometer (the view field area with a 20x objective would be 1.1 mm). Immunochemistry of TRPV4 is described in the Supplementary Materials & Methods.

### Micropipette aspiration

Micropipettes were pulled from borosilicate glass capillaries (B100-75-10, Sutter instruments) using a micropipette puller (Model P-97, Sutter Instruments) and forged to the required diameter (~7 μm) using a micropipette forge (MF-900, Narishige, Japan). To prevent non-specific adhesion between the capillary wall and the cell, micropipettes were filled with 3% BSA solution using a micropipette filler (Microfil^TM^, World Precision Instruments, Fl). The micropipette was then mounted on a micromanipulator (Eppendrof) and connected to water columns. Cells were first trypsinized, centrifuged and re-suspended in culture medium. A large drop of culture medium was placed on a hydrophobic glass cover slip and mounted on an inverted microscope (Leica). About 5 μl of the cell suspension was added to this drop of the culture medium. A single suspended cell was aspirated into the micropipette. Pressure was applied to the cell at a rate of 2 Pa/sec for 200 seconds (or max of 400 Pa). Images of the cell were captured every 2 seconds using a 63X dry objective (Leica). The shear modulus was calculated using the proportionality relation between applied suction pressure and aspirated length of the cell in the micropipette. The equation below describes the relation


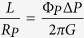


Here, L is the aspirated length, *R*_*P*_ the pipette radius, G the shear modulus, and Φ_*P*_ is a function of the ration of the pipette wall thickness to the pipette radius, Φ_*P*_ = 2.0–2.1 when the ration is equal to 0.2–1.0. In this case, based on the ratio value between 0.2–0.4 in different experiments, we chose Φ_*P*_ = 2.0.

### Intracellular Ca^2+^ measurement

Cells were loaded with 5 μM fura-2-AM (Molecular Probes) for 30 min at 37 °C in the measuring buffer contained 145 mM NaCl, 5 mM KCl, 1 mM MgCl_2_, 9 mM glucose, 0.2% BSA, 10 mM HEPES and 1 mM CaCl_2_ (pH 7.4 with NaOH), dislodged with Trypsin-EDTA before assaying for intracellular calcium concentration in a cuvette under constant, gentle stirring (1 ml final volume). 0.5% Triton-X was added to get R_max_ (as a positive control) and 20 mM EDTA was added to get R_min_ (as a negative control). Fluorescent emission was monitored at 510 nm with alternate excitation at 340 and 380 nm using a RF-5301PC Intracellular Ion Measurement System Spectrofluorophotometer (Super Ion Probe); Shimadzu Corporation. [Ca^2 + ^]_i_ was calculated based on Grynkiewicz’s two wavelength method^62^.

### Statistical methods

Cell-based assays and xenograft were analyzed by unpaired two-tailed Student’s t-test. Statistical analysis for other experiments done are described in the supplementary Materials and Methods.

## Additional Information

**How to cite this article**: Lee, W. H. *et al*. TRPV4 Regulates Breast Cancer Cell Extravasation, Stiffness and Actin Cortex. *Sci. Rep*. **6**, 27903; doi: 10.1038/srep27903 (2016).

## Supplementary Material

Supplementary Information

Supplementary Table S1

## Figures and Tables

**Table 1:**
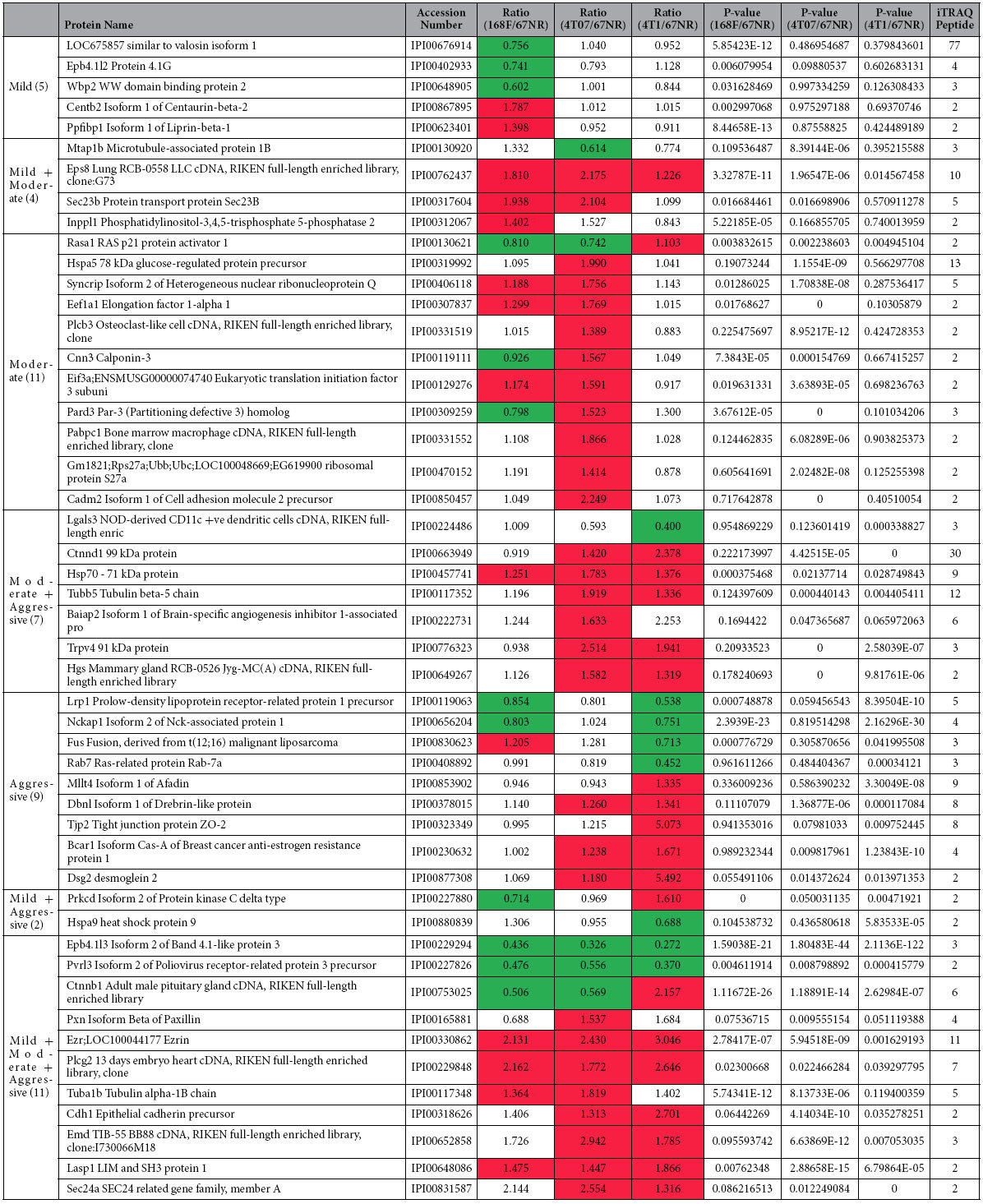
Relative levels of phosphoproteins across the cell lines in the Breast Cancer Metastasis model.

From 149 proteins detected, the proteins were subject to a 2-tier filtering system that eventually resulted in the 49 hits shown. The first filter was a 30% cut off to eliminate false positives. 30% is the technical variation of the analytical system used. The proteins that satisfied the 30% cut off (i.e., >1.3 or <0.77) were subject to a second filter whereby only iTRAQ ratios that are statistically significant i.e., p < 0.05 are selected. All statistical significance evaluation by Mann-Whitney test and Spearman correlation test were computed using Matlab®. Note: ‘NA’: Not applicable; where *p*-value = 0 means *p*-value is extremely small.
