# Supplementary material for: TRPV4 Regulates Breast Cancer Cell Extravasation, Stiffness and Actin Cortex
Source: Sci Rep. 2016 Jun 13;6:27903. doi: 10.1038/srep27903 (PMC4904279; doi:10.1038/srep27903)
Supplement: Supplementary Information [file srep27903-s1.pdf]

TRPV4 Regulates Breast Cancer Cell Extravasation, Stiffness and Actin Cortex

Wen Hsin Lee[1†], Lee Yee Choong[1†], Naing Naing Mon[1], SsuYi Lu[1], Qingsong Lin[2], Brendan Pang[3], Benedict Yan[4], Vedula Sri Ram Krishna[5], Himanshu Singh[6], Tuan Zea Tan [3], Jean Paul Thiery[1,3], Chwee Teck Lim[5,6], Patrick Boon Ooi Tan[7], Martin Johansson[8], Christian Harteneck[9] and Yoon Pin Lim[1,10,11*]

[1]Department of Biochemistry, Yong Loo Lin School of Medicine, National University of Singapore; [2]Department of Biological Sciences, Faculty of Science, National University of Singapore, [3]Cancer Science Institute of Singapore, [4]National University Hospital, Department of Laboratory Medicine; [5]Mechanobiology Institute, National University of Singapore; [6]Department of Biomedical Engineering, National University of Singapore; [7]Duke-NUS Graduate Medical School, [8]Respiratorius AB, Sweden; [9]Department of Pharmacology and Experimental Therapy, Institute of Experimental and Clinical Pharmacology and Toxicology, Eberhard Karls University Hospitals and Clinics, Tübingen, Germany; [10]NUS Graduate School for Integrative Sciences and Engineering, Singapore; [11]National University Cancer Institute, National University Health System.

† The authors contributed equally

* To whom all correspondence should be addressed: Yoon Pin LIM, Department of Biochemistry, MD4 #01-03D, 5 Science Drive 2, Singapore 117545. Tel: (65) 66011891; Fax: (65) 68739664.

E-mail: bchlyp@nus.edu.sg

Disclosure of Potential Conflicts of Interest: No potential conflicts of interest were disclosed.

Supplementary Figure 1

GQTSLHIAIER

LQWATTILDIER

HYVELLVAQGADVHAQA

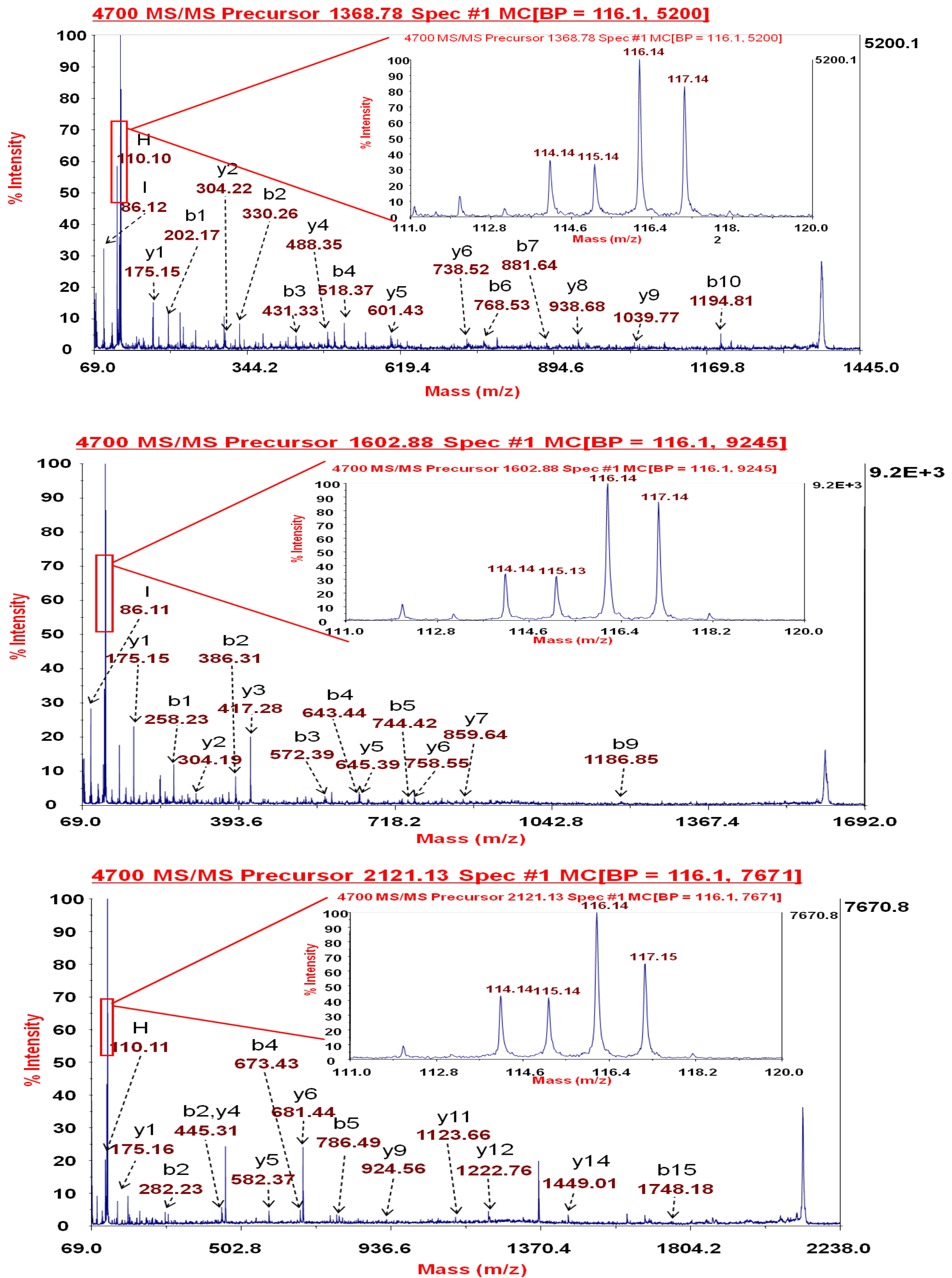

Supplementary Figure 2

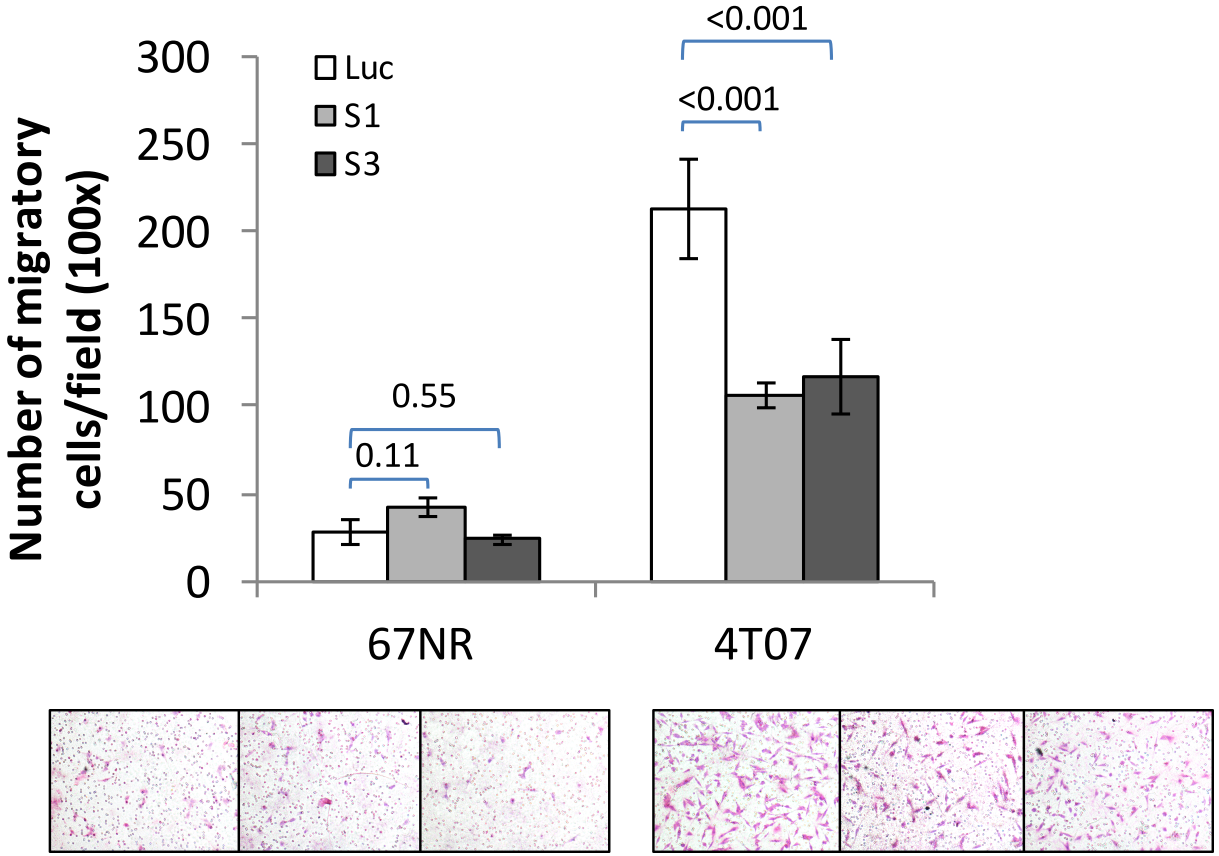

Supplementary Figure 3

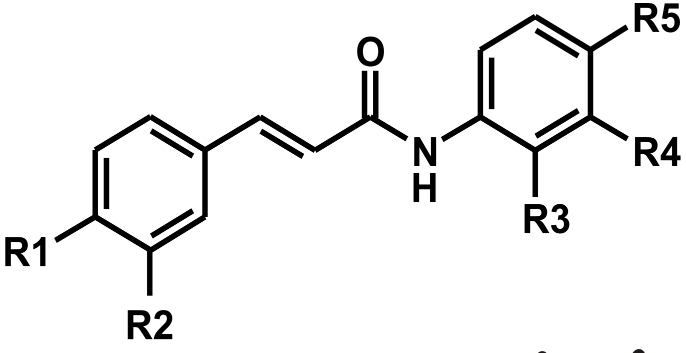

	R1	R2	R3	R4	R5	TRPC6	TRPM2	TRPM3	TRPV4
Tranilast	OCH_3	OCH_3	CO_2H	H	H	+/−	−	+/−	−
ACA	C_5H_{11}	H	CO_2H	H	H	++	++	++	++
ONO-RS-082	C_5H_{11}	H	CO_2H	H	Cl	+	++	++	++
RES019-29	C_5H_{11}	H	H	CO_2H	H	−	−	+/−	++

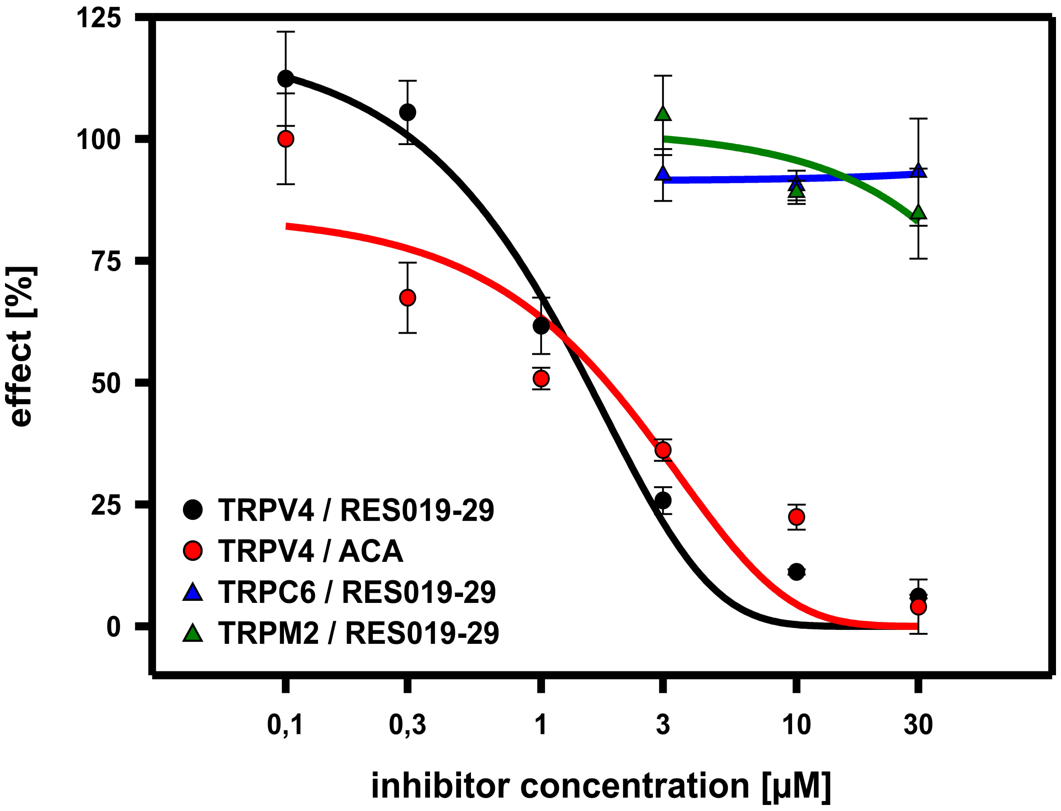

Supplementary Figure 4

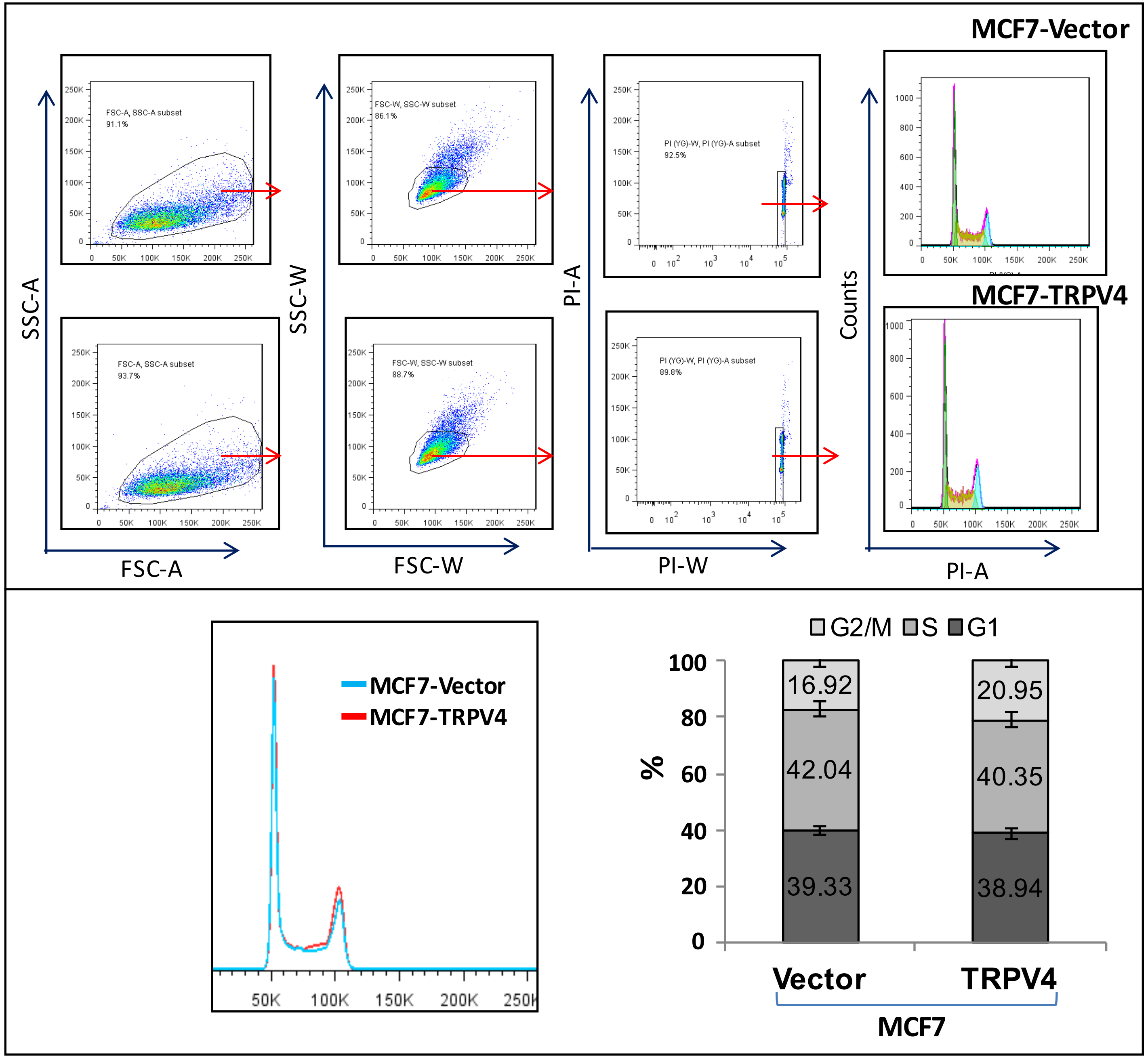

Summary of Analysis

INGENUITY SYSTEMS

Analysis Name:gene list - 2009-10-13 03:16 PM
Analysis Date:2009-10-13
IPA version:7.6

Analysis settings

View

Reference set: Ingenuity Knowledge Base (Genes Only)

Relationship to include: Direct and Indirect

Does not Include Endogenous Chemicals

Optional Analyses: My Pathways My List

Filter Summary:

Consider only molecules and/or relationships where

(species = Mammal) AND

(data sources = Ingenuity OR Protein-protein interactions)

Top Networks

ID	Associated Network Functions	Score
1	Cell Morphology, Cellular Development, Cancer	29
2	Cell Death, Neurological Disease, Cell-To-Cell Signaling and Interaction	16
3	Gene Expression, Infection Mechanism, Dermatological Diseases and Conditions	10
4	Cell Morphology, Cellular Assembly and Organization, Cancer	2
5	Nervous System Development and Function, Molecular Transport, Small Molecule Biochemistry	2

(c) 2000-2009 Ingenuity Systems, Inc. All rights reserved.

Supplementary Table 2

Top Bio Functions

Diseases and Disorders

Name	p-value	# Molecules
Cancer	2.40E-07 - 2.74E-02	29
Dermatological Diseases and Conditions	4.60E-05 - 2.74E-02	3
Skeletal and Muscular Disorders	7.93E-05 - 2.74E-02	10
Gastrointestinal Disease	8.55E-05 - 2.57E-02	18
Reproductive System Disease	1.12E-04 - 2.13E-02	12

Molecular and Cellular Functions

Name	p-value	# Molecules
Cellular Assembly and Organization	3.23E-08 - 2.74E-02	32
Cell Morphology	1.06E-07 - 2.74E-02	30
Cell-To-Cell Signaling and Interaction	2.40E-07 - 2.74E-02	18
Cellular Development	6.97E-07 - 2.74E-02	16
Cellular Movement	5.24E-06 - 2.74E-02	17

Physiological System Development and Function

Name	p-value	# Molecules
Reproductive System Development and Function	3.23E-06 - 2.35E-02	7
Tissue Development	3.54E-06 - 2.35E-02	17
Embryonic Development	1.54E-05 - 2.35E-02	9
Organ Development	4.60E-05 - 2.35E-02	5
Organ Morphology	4.60E-05 - 2.35E-02	6

(c) 2000-2009 Ingenuity Systems, Inc. All rights reserved.

INGENUITY SYSTEMS

Top Canonical Pathways

Name	p-value	Ratio
Leukocyte Extravasation Signaling	5.28E-11	11/189 (0.058)
Germ Cell-Sertoli Cell Junction Signaling	2.5E-10	10/155 (0.065)
Aldosterone Signaling in Epithelial Cells	6.45E-07	6/86 (0.07)
Actin Cytoskeleton Signaling	1.3E-05	7/232 (0.03)
VEGF Signaling	1.85E-05	5/91 (0.055)

Top Molecules

This analysis has no expression values.

(c) 2000-2009 Ingenuity Systems, Inc. All rights reserved.

Supplementary Table 2

Top My Lists

Name	p-value	Ratio

Top Pathways

Name	p-value	Ratio

Top Tox Lists

Name	p-value	Ratio
PPARα/RXR Activation	2.79E-02	3/166 (0.018)
Hypoxia-Inducible Factor Signaling	3.13E-02	2/70 (0.029)
Hepatic Cholestasis	9.97E-02	2/135 (0.015)
Oxidative Stress Response Mediated by Nrf2	1.94E-01	2/205 (0.01)
VDR/RXR Activation	2.64E-01	1/77 (0.013)

(c) 2000-2009 Ingenuity Systems, Inc. All rights reserved.

INGENUITY SYSTEMS

Supplementary Table 2

Top Tox Functions

Cardiotoxicity

Name	p-value	# Molecules
Cardiac Hypertrophy	5.43E-02 - 5.43E-02	2
Cardiac Stenosis	1.05E-01 - 1.05E-01	1
Cardiac Arteriopathy	1.00E00 - 1.00E00	3

Hepatotoxicity

Name	p-value	# Molecules
Liver Hepatomegaly	1.17E-03 - 1.17E-03	2
Liver Proliferation	3.89E-02 - 3.89E-02	1
Hepatocellular Carcinoma	1.08E-01 - 1.08E-01	2
Liver Cholestasis	2.12E-01 - 2.12E-01	1
Liver Damage	2.72E-01 - 2.72E-01	1

Nephrotoxicity

Name	p-value	# Molecules
Glomerular Injury	3.12E-02 - 3.12E-02	1
Renal Nephritis	3.89E-02 - 3.89E-02	1

(c) 2000-2009 Ingenuity Systems, Inc. All rights reserved.

Summary of Analysis

INGENUITY SYSTEMS

Analysis Name:gene list - 2009-10-13 03:16 PM
Analysis Date:2009-10-13
IPA version:7.6

Analysis settings

View

Reference set: Ingenuity Knowledge Base (Genes Only)

Relationship to include: Direct and Indirect

Does not Include Endogenous Chemicals

Optional Analyses: My Pathways My List

Filter Summary:
Consider only molecules and/or relationships where
(species = Mammal) AND
(data sources = Ingenuity OR Protein-protein interactions)

Top Networks

ID	Associated Network Functions	Score
1	Cell Morphology, Cellular Development, Cancer	29
2	Cell Death, Neurological Disease, Cell-To-Cell Signaling and Interaction	16
3	Gene Expression, Infection Mechanism, Dermatological Diseases and Conditions	10
4	Cell Morphology, Cellular Assembly and Organization, Cancer	2
5	Nervous System Development and Function, Molecular Transport, Small Molecule Biochemistry	2

(c) 2000-2009 Ingenuity Systems, Inc. All rights reserved.

Supplementary Table 2

Top Bio Functions

Diseases and Disorders

Name	p-value	# Molecules
Cancer	2.40E-07 - 2.74E-02	29
Dermatological Diseases and Conditions	4.60E-05 - 2.74E-02	3
Skeletal and Muscular Disorders	7.93E-05 - 2.74E-02	10
Gastrointestinal Disease	8.55E-05 - 2.57E-02	18
Reproductive System Disease	1.12E-04 - 2.13E-02	12

Molecular and Cellular Functions

Name	p-value	# Molecules
Cellular Assembly and Organization	3.23E-08 - 2.74E-02	32
Cell Morphology	1.06E-07 - 2.74E-02	30
Cell-To-Cell Signaling and Interaction	2.40E-07 - 2.74E-02	18
Cellular Development	6.97E-07 - 2.74E-02	16
Cellular Movement	5.24E-06 - 2.74E-02	17

Physiological System Development and Function

Name	p-value	# Molecules
Reproductive System Development and Function	3.23E-06 - 2.35E-02	7
Tissue Development	3.54E-06 - 2.35E-02	17
Embryonic Development	1.54E-05 - 2.35E-02	9
Organ Development	4.60E-05 - 2.35E-02	5
Organ Morphology	4.60E-05 - 2.35E-02	6

(c) 2000-2009 Ingenuity Systems, Inc. All rights reserved.

INGENUITY SYSTEMS

Supplementary Table 2

Top Canonical Pathways

Name	p-value	Ratio
Leukocyte Extravasation Signaling	5.28E-11	11/189 (0.058)
Germ Cell-Sertoli Cell Junction Signaling	2.5E-10	10/155 (0.065)
Aldosterone Signaling in Epithelial Cells	6.45E-07	6/86 (0.07)
Actin Cytoskeleton Signaling	1.3E-05	7/232 (0.03)
VEGF Signaling	1.85E-05	5/91 (0.055)

Top Molecules

This analysis has no expression values.

(c) 2000-2009 Ingenuity Systems, Inc. All rights reserved.

Supplementary Table 2

Top My Lists

Name	p-value	Ratio

Top Pathways

Name	p-value	Ratio

Top Tox Lists

Name	p-value	Ratio
PPARα/RXR Activation	2.79E-02	3/166 (0.018)
Hypoxia-Inducible Factor Signaling	3.13E-02	2/70 (0.029)
Hepatic Cholestasis	9.97E-02	2/135 (0.015)
Oxidative Stress Response Mediated by Nrf2	1.94E-01	2/205 (0.01)
VDR/RXR Activation	2.64E-01	1/77 (0.013)

(c) 2000-2009 Ingenuity Systems, Inc. All rights reserved.

INGENUITY SYSTEMS

Supplementary Table 2

Top Tox Functions

Cardiotoxicity

Name	p-value	# Molecules
Cardiac Hypertrophy	5.43E-02 - 5.43E-02	2
Cardiac Stenosis	1.05E-01 - 1.05E-01	1
Cardiac Arteriopathy	1.00E00 - 1.00E00	3

Hepatotoxicity

Name	p-value	# Molecules
Liver Hepatomegaly	1.17E-03 - 1.17E-03	2
Liver Proliferation	3.89E-02 - 3.89E-02	1
Hepatocellular Carcinoma	1.08E-01 - 1.08E-01	2
Liver Cholestasis	2.12E-01 - 2.12E-01	1
Liver Damage	2.72E-01 - 2.72E-01	1

Nephrotoxicity

Name	p-value	# Molecules
Glomerular Injury	3.12E-02 - 3.12E-02	1
Renal Nephritis	3.89E-02 - 3.89E-02	1

(c) 2000-2009 Ingenuity Systems, Inc. All rights reserved.

INGENUITY SYSTEMS

Supplementary Table 3

Distribution of nodules size (mm)

Control(n=60)		Knockdown(n=12)
0.19	0.296	0.185
0.19	0.093	0.123
0.44	0.093	0.0125
0.074	0.137	0.148
0.074	0.37	0.185
0.19	0.137	0.185
0.093	0.185	0.296
0.0123	0.37	0.093
0.148	0.185	0.185
0.185	0.737	0.248
0.296	0.185	0.137
0.19	0.074	0.185
0.074	0.148	
0.075	0.37	
0.0925	0.296	
0.0925	0.248	
0.19	0.44	
0.44	0.0074	
0.123	0.333	
0.296	0.093	
0.148	0.185	
0.37	0.148	
0.148	0.37	
0.185	0.093	
0.148	0.137	
0.074	0.248	
0.074	0.137	
0.148	0.137	
0.093	0.074	
0.25	0.148	

Supplementary Table 3

IHC:TRPV4 on lung sections from tail vein injection with 4T1 cells – Luc control versus S1+S3 knockdown [illegible]

Groups	Nodules number	TRPV4
Luc (n=22)	1	3+
	2	3+
	3	3+
	4	3+
	5	3+
	6	3+
	7	3+
	8	3+
	9	3+
	10	3+
	11	3+
	12	3+
	13	3+
	14	3+
	15	3+
	16	1+
	17	1+
	18	1+
	19	3+
	20	1+
	21	2+
	22	3+
S1+S3 (n=5)	1	1+
	2	0
	3	1+
	4	2+
	5	0

Supplementary Table 4

	Ctrl	Pressure 1	Pressure 2
Dec	Cell1	NA	NA
	cell2	46	184
	cell3	35	140
	cell4	46	184
	cell5	33	132
	cell6	41	164
	cell7	64	256
	cell8	53	212
	cell9	45	180
	cell10	42	168
	cell12	48	192
	cell13	NA	NA
	cell14	67	268
	cell15	35	140
Feb	cell1	49	196
	cell2	37	148
	cell3	65	260
	cell5	44	176
	cell6	45	180
	cell7	62	248
	cell8	61	244
	cell9	41	164
	cell10	NA	NA
			191.8

	S1	Pressure 1	Pressure 2
Dec	cell1	NA	NA
	cell2	NA	NA
	cell3	NA	NA
	cell4	NA	NA
	cell5	NA	NA
	cell6	84	336
	cell7	65	260
	cell8	53	212
	cell9	NA	NA
	cell10	NA	NA
	cell2	NA	NA
	cell3	NA	NA
	cell4	83	332
	cell5	NA	NA
	cell6	NA	NA
	cell7	98	392
	cell8	NA	NA
	cell9	NA	NA
	cell10	61	244
	cell11	50	200
			282.28571

	S3	Pressure 1	Pressure 2
Dec	cell1	NA	NA
	cell2	NA	NA
	cell3	80	320
	cell4	NA	NA
	cell5	NA	NA
	cell6	NA	NA
	cell7	50	200
	cell8	45	180
	cell9	NA	NA
	cell10	NA	NA
	cell2	NA	NA
	cell3	NA	NA
	cell4	NA	NA
	cell5	NA	NA
	cell6	63	252
	cell7	NA	NA
	cell8	NA	NA
	cell9	NA	NA
	cell10	62	248
			240

Supplementary Table 4

	Ctrl	S1	S3
Percentage of Cells Blebs (%)	87.5	33.3	25.0
Avg. Pressure at which bleb develops(Pa)	191.43	282.29	240.00

NA: no blebs formed

Pressure 1 - when bleb begins

Pressure 2 - when bleb reaches the mark

	Luc	S1	S3
Avg. Pressure (Pa)	191.43	282.29	240.00
SEM	9.07	27.2	24.3
**P-value (2-tailed)**		0	0.036

	**Percentage o**	**Std. Error Mea**	**P-value (2-tailed)**
Luc	87.5	6.8959661	
S1	33.3	10.540926	0.0000
S3	25.0	9.9339927	0.0000

Supplementary Legends

Fig S1 - The MS/MS spectra of the 3 TRPV4 iTRAQ peptides detected

Figure shows the relative intensity of the iTRAQ reporter ions derived from TRPV4 across the cell lines in BCM model as well as the sequence of the peptides detected.

Fig S2 – Effects of *TRPV4* knockdown on the migration ability in 67NR and 4T07 cells

Upper panel: 48 hours post siRNA transfection, number of migratory cells with siRNA control or *TRPV4* siRNA were counted. Lower panel: representative images (100x) were shown.

Fig S3 - Development of the TRPV4-selective Inhibitor RES019-29

(a) Based on the finding that ACA represents a pan TRP channel blocker, a TRPV4-selective inhibitor was developed. (b) The selectivity is show by the concentrations response curves provided. The data were generated using FLIPR^{Tetra}. Data from a representative experiment show the effect of RES019-29 on TRPC6-, TRPM2- and TRPV4-mediated calcium entry upon hyperforin (10 µM), hydrogen peroxide (5 mM) and water (changing osmolarity from 300 mosmol/l to 225 mosmol/l) application, respectively. Additionally, the effect of ACA on TRPV4 activity is shown in a concentration-dependent manner. The data were calculated from one experiment of at least two experiments performed in quadruplicate per concentration and TRP channel.

Fig S4 - Effects of TRPV4 expression on the cell cycle of MCF7 stable clones.

Upper panel: After gating on the live cells, single cells were gated using width and area parameters. Lower panel: the parameter histogram (in lower panel) was used to determine the percentages of cells in G1, S,and G2M phases.

Table S1- Protein-peptide summary for iTRAQ-based phosphoproteomics

Raw data showing the iTRAQ ratios and peptides used for relative quantification of phosphoproteins across the BCM model

Table S2 - Ingenuity Pathways Analysis

Summary of statistical analyses of the association of 49 phosphoproteins that displayed statistically significant difference in amounts across the BCM model with top network, function and canonical pathways

Table S3 - Tail vein injection studies

Tabulation of the number of metastatic nodules in mouse model and the IHC scores of TRPV4 in metastatic nodules.

Table S4 - Micropipette aspiration studies

Tabulation of the raw data on the pressure required for blebs to form and complete in control and TRPV4-knocked down cells

Supplementary Materials and Methods

Reagents

Agarose-conjugated or unconjugated anti-mouse and anti-rabbit IgG (whole molecule) used for immunoprecipitation and immunoblotting were purchased from Sigma Chemical Co. (St. Louis, MO). Horseradish peroxidase-conjugated PY20H mouse monoclonal anti-phosphotyrosine antibodies, anti-EPS8 and anti-PXN antibodies were from BD Biosciences (San Jose, CA, USA); PSCD2 antibody was from Proteintech Group, Inc. (Chicago, IL); SEC23B antibodies were from Abcam (Cambridge, U.K.); FUS and TRPV4 polyclonal antibodies were from Santa Cruz (Santa Cruz, CA); Total-Cofilin, phospho-Cofilin, Total VASP, phosphor-VASP and E-cadherin antibodies were from Cell Signaling Technology (Danvers, MA) and Enhanced chemiluminescence (ECL) detection kit was purchased from General Electric Healthcare, Bio-Sciences (Uppsala, Sweden), prestained molecular weight markers were from Bio-Rad (Hercules, CA), protease inhibitors cocktail were from Roche (Mannheim, Germany), and PolyVinylidene DiFluoride (PVDF) membranes were from Millipore (Bedford, MA). Sodium Orthovanadate and 3% (0.9 M) Hydrogen Peroxide were purchased from Sigma Aldrich (St Louis, MO).

The Triton-X was purchased from Sigma Chemical (St Louis, MO). Protease & phosphatase inhibitor cocktails were from Roche (Nutley, CA). Tris-base and EDTA were from First Base Laboratories Sdn Bhd (Selangor Darul Ehsan, Malaysia). JetPRIME™ transfection reagent was supplied by Polyplus-transfection Inc. (New York, USA).

LC-MS/MS of 4G10-purified phosphoproteins – Detection and Relative Quantification

For MALDI analysis, the iTRAQ labeled peptide mixture was separated using an Ultimate^{TM} LC system (Dionex-LC Packings) equipped with a Probot^{TM} MALDI spotting device. The iTRAQ peptide mixture was first captured with a 0.3 × 1 mm trap column (3 µm C_{18} PepMap^{TM}, 100 Å) (Dionex-LC Packings) and washed with 0.05 % TFA followed by gradient separation with a 0.2 × 50 mm reverse-phase column (Monolithic PS-DVB) (Dionex-LC Packings). The mobile phase A and B used were 98 % H_2O, 2 % ACN with 0.05 % TFA and 80 % H_2O, 20 % ACN and 0.04 %TFA respectively. The gradient elution step was 0-60 % mobile phase B in 20 min, with a flow rate of 2.7 µl/min. The LC fractions were mixed with MALDI matrix solution (7 mg/ml α-cyano-4-hydroxycinnamic acid and 130 µg/ml ammonium citrate in 75% ACN) in a flow rate of 5.4 µl/min through a 25 nl mixing tee (Upchurch Scientific) before spotting onto 192-well stainless steel MALDI target

plates (Applied Biosystems, Foster City, CA, USA) using a Probot Micro Fraction collector (Dionex-LC Packings), with a speed of 5 sec per well. MALDI target plates were analyzed using an ABI 4700 Proteomics Analyzer MALDI-TOF/TOF mass spectrometer (Applied Biosystems, Foster City, CA, USA) operating in a result independent acquisition mode. Typically 1000 shots were accumulated for each well of sample. MS/MS analyses were performed using nitrogen, at collision energy of 1 kV and a collision gas pressure of $\sim 1 \times 10^{-6}$ Torr. For precursor ions with signal to noise (s/n) ratio greater or equal to 100, 6,000 shots were combined for each spectrum. For the precursors with s/n ratio between 50 and 100, 10,000 shots were accumulated. GPS Explorer software version 3.5 (Applied Biosystems) was used to create and search files with the MASCOT search engine version 2.1 (Matrix Science, Boston, MA) for protein identification and quantification. The International Protein Index mouse database version 3.39 (www. ebi.ac.uk/IPI/IPIhelp.html, 52777 entries) was used. The search was restricted to tryptic peptides and one missing cleavage was allowed. N-terminal iTRAQ labeling, iTRAQ labeled-lysine and Cysteine methanethiolation were chosen as fixed modifications and methionine oxidation as variable modifications. Precursor error tolerance and MS/MS fragment error tolerance were set to 100 ppm and 0.3 Da, respectively. The maximum peptide rank was set at 2 and the minimum ion score CI% of peptide was set at 85. iTRAQ ratios are calculated based on the relative cluster areas of the iTRAQ reporter fragment peaks (114, 115, 116 and 117), with 114 peaks as references. The normalization option was not executed since this study concerns chemically-induced protein phosphorylation that can take place to different extends in various cells. To estimate the false-discovery rate (FDR) in the dataset obtained, a randomized database (52777 entries) was generated using all the sequence entries from the International Protein Index mouse database version 3.39, with a Pearl script downloaded from Matrix Science. FDR was <1%. During the course of the study, IPI database became obsolete and UniProt IDs for the IPI hits were used instead during data presentation.

Real Time PCR

Cells were grown in a 10-cm tissue culture dish until 80–90% confluence (1×10^7 exponentially dividing cells). Total RNA for each cell line was extracted using the Qiagen RNasy isolation kit (Qiagen, West Sussex, UK) according to the manufacturer's protocol. For first strand cDNA synthesis, 2 μg of RNA were reverse transcribed using the following conditions: 1x avian myeloblastosis virus reverse transcriptase reaction buffer (Promega), 30 units of avian myeloblastosis virus reverse transcriptase (Promega), 40 units RNasin ribonuclease inhibitor (Promega), 1 mM dNTP mixture (Invitrogen), 1 μg of oligo(dT) (Invitrogen). Each RT-PCR was

performed in triplicates and pooled. The integrity of the pooled cDNA was assessed by PCR amplification with a human control gene, ribosomal *18S* and *Gadph*. Negative control (without cDNA template) was also included to check for contaminating cDNA and genomic DNA. Evaluation of the gene expression profile of the *TRPV4* was performed using the TaqMan Gene Expression Assay and gene-specific primers: mouse probes (*Trpv4* Mm00499025_m1, and *Gadph* Mm99999915_g1) from Applied Biosystems and human probes (*TRPV4* Hs.PT.39a.22214856.g and *18S* Hs.PT.56a.985962) from Integrated Technologies Inc (Iowa, USA). Sample-specific PCR mixture was loaded in triplicates onto the microplate at 50 ng of cDNA/well and analyzed using the ABI 7300 system. Quantification of gene expression relative to the "normal" cell line or control samples was then done using the comparative *CT* method as described in the manufacturer's manual.

Calculation of G-actin/F-actin Ratio

To determine globular (G-actin) to filamentous actin (F-actin) ratio, the G-actin/F-actin *in vivo* assay kit from Cytoskeleton (Denver, CO) was used according to the instructions of the manufacturer. Briefly, cells at 80% confluency were lysed, and the lysate was centrifuged at 100,000 × *g* for 1 h at 37 °C. The supernatants contained G-actin, whereas the pellet contained F-actin. As a control, cell lysates are either treated with F-actin enhancing solution (phalloidin) or F-actin depolymerization solution (cytochalasin) before ultracentrifugation. The amount of G- and F-actin was analyzed by Western blotting using an anti-actin-specific antibody.

***In Vitro* Cell-Based Assays**

For transendothelial migration assays, 100,000 HUVEC cells were cultured on each 8 μm pore size insert (Cell Biolabs Inc., San Diego, CA) for 48 h. 2 x 10^5 of the overnight serum starved transfected cells were added onto the monolayer of the HUVEC cells and the insert was then transferred to a new plate containing fresh medium with 10% fetal bovine serum (FBS). Assay was performed at 37 ˚C for 8 h. Non-migrating cells at the top were removed, whereas cells that have migrated to the bottom of the membrane were first dissociated from the membrane, then lysed and quantified using CyQuant GR fluorescent dye at 480 nm/520 nm. Experiments were performed with three technical replicates and independently validated across two biological experiments.

Cell proliferation assay was performed using the CellTiter 96® Aqueous One Solution Non-Radioactive Cell Proliferation MTS Assay Reagent from Promega (San Luis, CA). Briefly, 2000-5000 cells per well were seeded in 100 µl culture medium in a 96-well plate on Day 0 and the cell growth was monitored until the fourth

Day. Twenty µl of the MTS reagent was added to each well using a multichannel pipet and mixed by swirling the plate. The absorbance was measured within an hour on a plate reader (Tecan) at 490 nm. All experiments were performed with three technical replicates and across three independent biological experiments.

For wound healing assays, cells were seeded onto 6-well plate and grown until a confluent monolayer. A wound was incised onto the cell monolayer with a p200 pipet tip. The cells were washed once with growth medium to remove the cell debris and to smoothen the edge of the scratch and then replaced with fresh growth medium. The cells were incubated at 37 °C and cell migration was monitored up to 24 h. Using a phase-contrast microscope, images were captured at 0, 8, 16 and 24 h after scratch. The relative width of the scratch was measured quantitatively using Photoshop. The extent of gap closure over time was determined as the rate of cell migration. Experiments were performed with three technical replicates and independently validated across three biological experiments.

For chemotaxis assay, 50,000 serum-starved cells were added to the top chambers of the 96-well trans-well plate lined with polycarbonate membrane chambers (8 µm pore size, Cell Biolabs Inc., San Diego, CA). Medium containing 10% fetal bovine serum (FBS) was added to the bottom chambers as a chemoattractant. Cells were allowed to migrate for 4 h. Non-migratory cells at the top chambers were removed, while cells that have migrated to the bottom chambers were first fixed with methanol for 10 min and then stained with Hematoxylin&eosin stain. The number of migrating cells was determined by microscope at 100x magnification on each membrane and calculated the mean number of cells per field. Experiments were done in triplicates and independently validated across three biological experiments.

For invasion assay, 100,000 overnight serum-starved cells were added to the top chambers of the 24-well trans-well plate (8 µm pore filters, BD Biosciences, Bedford, MA USA) lined with polycarbonate membrane coated with a uniform layer of matrigel (BD Biosciences, Bedford, MA USA). Medium containing 10% fetal bovine serum (FBS) was added to the bottom chambers. Assay was performed at 37 ˚C for 24 h. Non invasive cells at the top were removed, whereas cells that have invaded to the under-side of the membrane were first fixed with methanol for 10 min and then stained with Hematoxylin&eosin stain. Experiments were done in triplicates and independently validated across three biological experiments.

Cell Cycle Analysis

1 x 10^4 of the cells were fixed with ice-cold 70% ethanol on ice for 30 min, and then washed with PBS to remove the ethanol. Cell pellet were resuspended in propidium iodide (50 µg/ml) / RNaseA (100 µg/ml)

solution for 10 min. Samples were analyzed on the flow cytometry (BD™ *LSR II*) at a low flow rate under 400 events/second. Cell cycle distribution was determined by the FlowJo software.

Immunohistochemistry

Formalin fixed, paraffin embedded sections were warmed in a 60 °C oven, dewaxed in three changes of histoclear and passaged through graded ethanol (100%, 95%, and 70%) before a final wash in double distilled H_2O. Antigen retrieval was performed using the Target Retrieval Solution (DakoCytomation, Glostrup, Denmark) at 95 °C for 40 min. After quenching of endogenous peroxidase activity with 3% H_2O_2 for 10 min and blocking with 5% BSA for 30 min, sections were incubated at 4 °C for 24 h with antibody against TRPV4 at a 1:500 dilution. Detection was achieved with the Envision+/horseradish peroxidase system (DakoCytomation). All slides were counterstained with Gill's hematoxylin for 1 min, dehydrated, and mounted for light microscopic evaluation. All statistical tests were performed at 5% significance level with the statistical software SPSS 14.0 for Windows. All statistical analyses associated with clinical samples were done in R version 2.15.1 at 5% significance level unless otherwise stated (The R Foundation for Statistical Computing). Average IHC scores between lung tissue sections from SCID mice injected with ctrl and *Trpv4*-knockdown 4T1 cells, were compared by the two-sample t-test.

Transfection

For knockdown experiments, cells were seeded at 70-80% confluency in 60 mm dish in medium containing 10% FBS and 100U Penicillin/Streptomycin one day before transfection and transfected with 200 nM siRNA and 10 µl jetPRIME™ reagent (Polyplus Transfection Inc.) according to the manufacturer's instructions. Cells were harvested 48 h post-transfection. Mock transfections and non-specific siRNA duplexes were used as the negative controls. Cells were treated for 48 to 72 h to allow maximum knockdown, after which they were either harvested for Western blot analysis or used for functional assays.

Retroviral Constructs and Transduction of Breast Cancer Cells

The pBabe-TRPV4 vector was constructed by subcloning the V5-tagged 3' *TRPV4* cDNA fragment (amino acids 1-871 into pBabe (*Addgene,* Cambridge MA USA) upstream of SV40 immediate early promoter (IEP) and an antibiotic resistance gene (puromycin or neomycin) at *BmaHI and SalI cloning sites.* The Phoneix amphotropic retrovirus packaging cell line was transfected with retroviral vectors using JetPrime-mediated

PolyPlus transfection kit and supernatant was harvested after 48 hours. Unconcentrated retroviral supernatant, cleared of cell debris, was used to transduce the breast cancer cells lines (at ~70% confluence) by spinoculation (centrifugation at 1000 *g*, 25 °C, 2 h) in the presence of 5 µg/ml polybrene (Sigma-Aldrich, St. Louis, MO USA), and returned to culture afterwards. Viral supernatant was replaced with fresh growth medium next day. After 48-72 h post retroviral transduction, stable integrants were obtained through puromycin (2 µg/ml) or G418 (400 µg/ml) selection up to 2 weeks. The cell survival rate was at 40-60% after first round (3-5 days) of antibiotics selection.

Data Preprocessing of Affymetrix Microarray Gene Expression and Epithelial-Mesenchymal Transition Score

Microarray gene expression data of ovarian cancer on Affymetrix U133A or U133Plus2 platforms were downloaded from Array Express and Gene Expression Omnibus (GEO). For this study, we included all of publicly available dataset at the time the analysis was initiated. Robust Multichip Average (RMA) normalization was performed on each dataset. The normalized data was combined and subsequently standardized using ComBat (1) to remove batch effect. The ovarian cancer panel consists of 1,538 human ovarian carcinoma from 16 cohorts, including GSE3149 (n = 146), GSE9891 (n = 285), GSE2109 (n = 214), TCGA (The Cancer Genome Atlas; n = 406), Oslo Cohort (n=26), GSE6008 (n = 99), E-MEXP-935 (n = 27), GSE10971 (n = 13), GSE14001 (n = 20), GSE14407 (n = 12), GSE14764 (n = 80), GSE18520 (n = 53), GSE19352 (n = 20), E-MEXP-1085 (n = 43), GSE15578 (n = 4), GSE12172 (n = 90). Our gastric cancer gene expression data was previously deposited in the NCBI Gene Expression Omnibus (GEO) database: Affymetrix U133P GSE15460 (2). Preprocessing was performed as described previously and data were presented in log2.

Computation of EMT score is as described previously (3). An Epithelial-Mesenchymal Transition (EMT) signature was developed by comparing expression profiles of *CDH1* with *CDH2* expressing ovarian carcinomas cell lines using Binary Regression method (4). The BinReg ovarian cancer EMT signature was then applied to predict the EMT status of breast cancer tumors. Subsequently, the top 5% (~100 samples) with the highest probabilities for epithelial or mesenchymal phenotype were used to obtain the epithelial or mesenchymal specific gene list for the breast cancer tumor (EMT signature) using Significance Analysis of Microarray (SAM) *q*-value =0 and ROC value of 0.85. To obtain EMT score of a breast cancer tumor, the enrichment scores of the breast cancer tumor-specific epithelial and mesenchymal gene list were computed using Single Sample GSEA (5). The EMT score is defined as the difference in enrichment scores of the mesenchymal signature from

epithelial signature. Hence, EMT score ϵ[-1.0,+1.0], where EMT score = +1.0 indicates fully mesenchymal, and EMT score = -1.0 indicates fully epithelial.

All statistical significance evaluation by Mann-Whitney test and Spearman correlation test were computed using Matlab®. Log-rank test, Kaplan-Meier and dot plots were performed in Graphpad Prism.

1. Johnson, W. E., Li, C., and Rabinovic, A. (2007) Adjusting batch effects in microarray expression data using empirical Bayes methods. *Biostatistics* **8**, 118-127
2. Ooi, C. H., Ivanova, T., Wu, J., Lee, M., Tan, I. B., Tao, J., Ward, L., Koo, J. H., Gopalakrishnan, V., Zhu, Y., Cheng, L. L., Lee, J., Rha, S. Y., Chung, H. C., Ganesan, K., So, J., Soo, K. C., Lim, D., Chan, W. H., Wong, W. K., Bowtell, D., Yeoh, K. G., Grabsch, H., Boussioutas, A., and Tan, P. (2009) Oncogenic pathway combinations predict clinical prognosis in gastric cancer. *PLoS Genet* **5**, e1000676
3. Akalay, I., Janji, B., Hasmim, M., Noman, M. Z., Andre, F., De Cremoux, P., Bertheau, P., Badoual, C., Vielh, P., Larsen, A. K., Sabbah, M., Tan, T. Z., Keira, J. H., Hung, N. T., Thiery, J. P., Mami-Chouaib, F., and Chouaib, S. (2013) Epithelial-to-mesenchymal transition and autophagy induction in breast carcinoma promote escape from T-cell-mediated lysis. *Cancer Res* **73**, 2418-2427
4. Gatza, M. L., Lucas, J. E., Barry, W. T., Kim, J. W., Wang, Q., Crawford, M. D., Datto, M. B., Kelley, M., Mathey-Prevot, B., Potti, A., and Nevins, J. R. (2010) A pathway-based classification of human breast cancer. *Proc Natl Acad Sci U S A* **107**, 6994-6999
5. Verhaak, R. G., Hoadley, K. A., Purdom, E., Wang, V., Qi, Y., Wilkerson, M. D., Miller, C. R., Ding, L., Golub, T., Mesirov, J. P., Alexe, G., Lawrence, M., O'Kelly, M., Tamayo, P., Weir, B. A., Gabriel, S., Winckler, W., Gupta, S., Jakkula, L., Feiler, H. S., Hodgson, J. G., James, C. D., Sarkaria, J. N., Brennan, C., Kahn, A., Spellman, P. T., Wilson, R. K., Speed, T. P., Gray, J. W., Meyerson, M., Getz, G., Perou, C. M., and Hayes, D. N. (2010) Integrated genomic analysis identifies clinically relevant subtypes of glioblastoma characterized by abnormalities in PDGFRA, IDH1, EGFR, and NF1. *Cancer Cell* **17**, 98-110
